# Metal-organic frameworks: Drug delivery applications and future prospects

**DOI:** 10.5599/admet.2057

**Published:** 2023-11-17

**Authors:** Prachi Mhettar, Niraj Kale, Jidnyasa Pantwalawalkar, Sopan Nangare, Namdeo Jadhav

**Affiliations:** 1 Department of Pharmaceutics, Bharati Vidyapeeth College of Pharmacy, Kolhapur 416013 Maharashtra State, India; 2 Department of Pharmaceutics, Dr. D. Y. Patil Institute of Pharmaceutical Sciences & Research, Pimpri, Pune 411018, Maharashtra State, India; 3 Department of Pharmaceutical Chemistry, H. R Patel Institute of Pharmaceutical Education and Research, Shirpur 425405, Maharashtra State, India; 4 Krishna Vishwa Vidyapeeth, Krishna Institute of Pharmacy Karad- 415539, Maharashtra State, India

**Keywords:** stimuli-responsive, drug loading, formulation development

## Abstract

**Background and purpose:**

Metal-organic frameworks (MOFs) have gained incredible consideration in the biomedical field due to their flexible structural configuration, tunable pore size and tailorable surface modification. These inherent characteristics of MOFs portray numerous merits as potential drug carriers, depicting improved drug loading, site-specific drug delivery, biocompatibility, biodegradability, etc.

**Review approach:**

The current review article sheds light on the synthesis and use of MOFs in drug delivery applications. In the beginning, a brief overview of the key components and efficient fabrication techniques for MOF synthesis, along with its characterization methods, have been presented. The MOFs-based formulations have been critically discussed. The application of the design of experiments (DoE) approach to optimize MOFs has been elucidated. The MOFs-based formulations, especially the application of stimuli-responsive MOFs for site-specific drug delivery, have been deciphered. Along with drug release kinetic models, several administration methods for MOFs have also been enunciated. Subsequently, MOFs as future potential drug carriers have been elaborated.

**Key results and conclusion:**

Recently, MOFs have emerged as versatile drug delivery carriers possessing customization potential and meeting the needs of spatio-temporal drug delivery. Researchers have devised several environment-friendly approaches for MOF construction and surface modification. Owing to stimuli-responsive potential, MOFs have demonstrated their prominent therapeutic efficacy via several routes of administration. The numerous benefits of MOFs would certainly open up a new vista for its novel drug delivery applications.

## Introduction

Conventional drug delivery systems (DDS) are available in a range of dosage forms, including tablets, capsules, granules, ointments, syrups for oral administration, and suppositories or solutions for intravenous administration. Conventional DDS fail to sustain the release owing to an array of drawbacks, such as frequent administration, a maximum dose demand, inadequate availability at target sites, fluctuations in plasma drug level, challenges in drug level monitoring, poor bioavailability concern, serious adverse/undesirable effects, and premature elimination from the body [1]. With the continued advancement in the field of nanotechnology, it has taken a lot of work to create nanocarriers for controlled drug release, which will increase therapy effectiveness while lowering adverse effects [2]. Metal-organic frameworks (MOFs) are porous compounds comprising metal ions and coordinated organic linkers, first reported in 1989 [3]. MOFs can be designed in a variety of ways to have elevated porosity structures owing to the adaptable mix of metal inorganic centres and organic ligands, which distinguishes them from other nanostructures. Considering their distinctive qualities, including huge porosity, an array of porous designs, huge surface areas extended up to 8000 m^2^ g^-1^, and customizable frameworks, these materials have attracted significant limelight as an emergent and potential group of porous materials for a variety of applications [4,5]. Considering their numerous benefits and broad applicability, the scientific community has paid a great deal of attention in the past 10 years to the investigation of novel approaches for MOF synthesis [6]. Essentially, these conjugates are made up of two ingredients: metal ions and organic linkers. Most synthesis methods include heating a mixture of bridging ligand-metal salts dissolved in a solvent solution utilising microwaves, electrical heating equipment, ultrasonic radiation, mechanical energy, *etc.* [7].

In recent times, MOFs have been thoroughly investigated as potential drug delivery system due to their formidable structural attributes [8]. Currently, the Cambridge Structural Database (CSD) has roughly synthesised 99,075 MOF and MOF-type compounds. As evidenced by the expanding number of studies examining their uses, MOFs have drawn rising interest for diverse applications, such as intriguing and burgeoning porous hybrid materials [9]. In addition to MOFs' beneficial characteristics for drug delivery applications, adding a guest material or encasing a MOF in a host material has been speculated to boost a MOF's effectiveness, resulting in MOF-composites [10]. With the ongoing advancement in MOFs, stimuli-responsive MOFs are a prominent candidate for targeted delivery because of their modulation in the local physiological system, which responds to the change in a milieu of the normal physiological system. A physical or chemical alteration in the physiological microenvironment can release drugs to the targeted site [11].

Thus, the MOFs could be the choice for future carriers for drug delivery applications.

## Synthesis of MOFs

The synthesis of MOFs is influenced by a variety of variables, including reaction duration and temperature, solvent type, the type of metal ions and organic linkers, structural features, anion-cation existence, and crystallisation kinetics, that may result in nucleation and crystal growth. MOFs are typically synthesized in the liquid phase by combining mixtures of the linkers and the metal ions. The solvent is chosen according to its reaction capacity, solubility, and redox capacity. The thermodynamics and energy of activation of each reaction are significantly influenced by the solvent. Despite challenges with single-crystal formation, solid-state synthetic techniques have occasionally been employed. MOF crystals have frequently been grown via the gradual evaporation of the resulting solution [12]. MOFs are synthesised by various methods, such as conventional methods, microwave-assisted synthesis, dry gel conversion, ionothermal synthesis, diffusion synthesis, microfluidic methods, reverse-phase microemulsions, electrochemical synthesis, mechanochemical methods, and sonochemical methods [13]. Along with their peculiarities, the limitations of such methods are briefly discussed below in Table 1.

**Table 1:** Methods of MOF synthesis along with their properties and limitations

Synthesis method	Properties	Ref.
Conventional	Conventional synthesis methodology entails a mixture of polar solvent and a metal precursor in sealed vessels at specified reaction conditions. According to reports, this method might produce crystalline MOFs with a higher output. This is supported by the higher solubility of precursors under high pressure and temperature. This method has several benefits, such as the fact that it can be used because it doesn't depend on a complicated reaction setup. Solvothermal synthesis can take several days and frequently calls for high pressure and temperature conditions. Consequently, safety concerns and time-consuming (expensive) issues prevent the large-scale manufacture of nano MOFS (nMOFs) by the hydro/solvothermal approach. Additionally, these syntheses frequently call for hazardous solvents like DMF.	[14]
Microwave	For the rapid synthesis of MOFs, the microwave method is extensively used. By exposing the reaction mixture to microwave radiation, MOFs are synthesized. The morphology of the ingredients can be altered by the MW approach, which also improves the performance of special MOF structures. The approach has many benefits, including monodispersed MOFs, high yield, minimal toxicity, and quick processing times.	[15,16]
Dry gel conversion	A gel precursor forms, and after being exposed to solvent vapour, it is transformed into a crystalline MOF. The MOF crystal growth is guided by solvent vapour, which serves as a template. The popularity of a recently created technique is attributed to its many benefits, including feasibility, high efficiency, high yield, minimal waste, reaction volume reduction, *etc*. The two MOFs were produced using water as the solvent medium instead of a traditional organic solvent like DMF, which is used in traditional synthesis techniques, making the process more environmentally friendly and economically viable.	[15,17]
Ionothermal	The strategy substitutes organic solvents and solvents like water with ionic liquids (ILs). The mixture of metal salts and linkers is dissolved in IL and heated in order to get MOFs. Due to their unique qualities, such as their excellent solvating properties (the ability to dissolve an array of metal salts and linkers), zero vapour pressure, high thermal stability, and ease of recycling, ILs have gained significant attention as a solvent.	[17]
Diffusion synthesis	The diffusion technique uses gas, liquid, and gel-phase diffusion. The center metal ions and organic linkers are dissolved in an incompatible solvent using the liquid-phase diffusion technique. Metal ions and organic ligands interact at the surface, forming MOF crystals. In the gel diffusion approach, the two divisions of a set of MOFs are obtained as gel phase crystals by mixing a solution containing metal ions and a gel component dispersed with organic ligands for a predetermined time. The volatile organic ligand solution is the solvent in the gas-phase diffusion procedure. MOFs develop as the reaction between the metal ion solution and the linker mixture is adequately reacted. A unique method that operates in mild reaction conditions makes it advantageous for the synthesis of sensitive MOFs.	[18,19]
Microfluidic	A microfluidic setup is used to synthesize MOFs. The syringe pumps are used for introducing reactant solutions via microchannels, while a mixing chamber is used to combine both of them. The MOF is subsequently produced by passing the combined solution through a reaction chamber. To facilitate the formation of the MOF, the reaction chamber is normally heated to a specified temperature. To meet the constantly rising commercial needs, it is crucial to investigate quick and sustainable MOF synthesis methods.	[20,21]
Reverse phase microemulsion	Researchers frequently use the reverse phase microemulsion technique for synthesizing uniform MOFs by introducing components to aqueous spherical that are reverse micelle-marinized for a reaction. Although it permits size control, the limited yield limits its usefulness.	[22]
Electrochemical	Electrochemical synthesis includes two approaches called the direct and indirect approach of MOF synthesis. Direct method based on the continuous anodic dissolution of metal ions into the organic linker-electrolyte combination to fabricate the MOFs. In comparison, the indirect method produces the intermediates via an electrochemical reaction employed to synthesis MOFs. Protic solvents are frequently used to prevent the breakdown of metals via complete utilization of linkers, which offer high yield and function under benign experimental conditions.	[23]
Mechanochemical	This approach of MOF synthesis uses metallic precursors and linking organic ligands. When these two components react chemically, coordination complexes are formed, and intramolecular interactions are reoriented. A mechanical deformation of intramolecular ties occurs before the chemical reaction and synthesis of the metal-organic complex. The current technique utilized mechanical pressure to directly grind reacting species with or without the use of a solvent. Mechanochemical milling is able to overcome the low melting point of the reactants and form the desired MOF structure. Also the solvents, which can be costly and dangerous for the environment, are not needed when using this method confirms as a green method of synthesis.	[24-26]
Sonochemical	The precursor solution interacts with ultrasonic waves (20-1000 kHz), leading to alternate compression and refraction areas and creating homogeneous nano-MOF (nMOF) via synthesis. This is a swift and environmentally friendly approach to creating MOFs. This process, which uses homogeneous and rapid nucleation, can result in substantially smaller particles and a shorter crystallization period than conventional synthesis methodologies.	[12,27]

## Characterization methods

**Figure 1.**
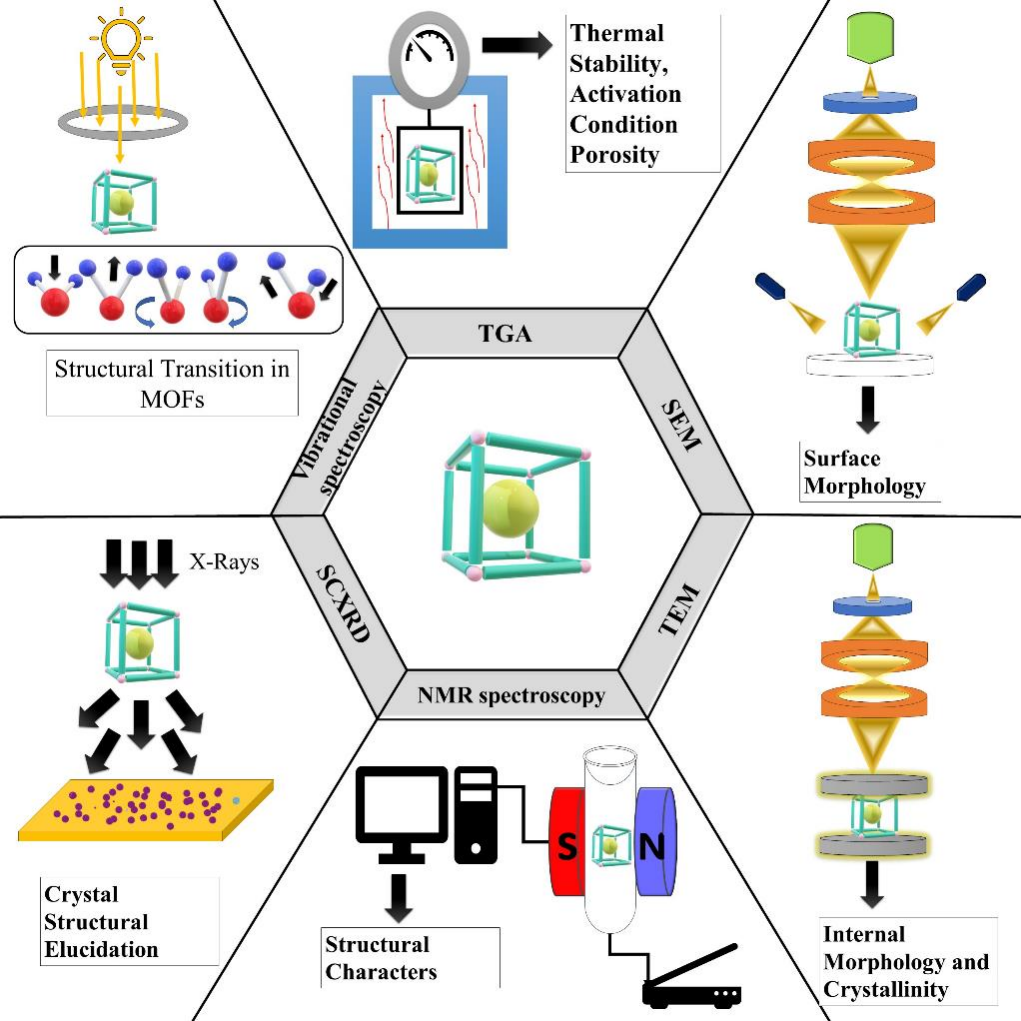
MOF characterization techniques

To compare and thoroughly describe the created MOFs' characteristics, including their size, shape, surface morphology, and thermal stability. The below-mentioned characterization techniques include TGA (thermogravimetric analysis), NMR (nuclear magnetic resonance), vibrational spectroscopy, SEM (scanning electron microscopy), PXRD (powder x-ray diffraction), SCXRD (single crystal X-ray diffraction), *etc*., along with characterising certain MOF properties as depicted in Figure 1 [28]. TGA, which analyses a sample's mass in relation to temperature, is a direct technique for examining the thermal stability of MOFs. Mass spectrometers and thermogravimetric analysers frequently work together.

As a result, it is able to identify the molecules responsible for the sample mass change as well as the temperature at which this mass shift occurs [29]. TGA is a useful tool for characterising MOFs that may be used to ascertain their thermal stability, activation conditions, and porosity. It has also been extensively used to quantify the compositional makeup of MOFs [30]. SEM is a crucial technique to analyse the varying surface-architecture attributes like size, surface morphology, and elemental configuration of MOFs [31]. This technique scans the surface of a solid sample with an electron beam with an intensity of almost 5 keV. The concentrated incident beam's electrons impact the specimen's surface and produce secondary electrons. These electrons are captured using a detector and used to generate the sample image [32]. In the transmission electron microscopy (TEM) experiment, a high-energy electron beam (200 keV) is focused on a thin sample (usually less than 200 nm) formed of a carbon grid where a drop sample has been evaporated. The electrons travelling through the sample, or being transmitted, are dispersed at multiple angles and then concentrated on a detector using a lens system to produce micrographs with great lateral spatial resolution. High magnification ranging from 50 to 10^6^ and the potential to furnish image and diffraction pattern information are big pluses of TEM. This is particularly important for MOF NPs because it demonstrates crystalline structure [32]. NMR spectroscopy is widely employed in the chemical sciences as a structural characterization tool. The utilisation of solid-state NMR spectroscopy (SSNMR) is required for the investigation of host-guest interactions in MOF-biomolecule systems [33]. SSNMR is an effective method for MOF characterisation since it provides details complementary to X-ray diffraction (XRD). Researchers are making innovative efforts in the three key aspects of SSNMR for MOF characterization: investigating the metal centre, focusing linker molecules, and investigating guests. MOFs have a lower density, and the inserted metals are frequently quadrupolar nuclei, rendering SSNMR identification problematic. Awhile back, researchers used SSNMR to investigate the structures of metal centres in prominent MOFs at a high magnetic field of 21.1 T, mentioning multiple critical topics such as (1) resolving chemically and crystallographically non-equivalent metal sites; (2) investigating the roots of disorder around metals; (3) refining local metal geometry; (4) probing the effects of activation and adsorption on the metal local environment; and (5) tracking in situ phase transitions in MOFs [34]. SCXRD has unquestionably been regarded as the most straightforward technique in the structural elucidation of crystallographic frameworks for conclusively clarifying the structure-property correlations. This handy tool can be used to directly see precise and comprehensive structural information such as atomic coordinates, bond angles, bond lengths, and atomic occupancies [35]. On the other hand, the size and quality of the crystal produced, as with XRD on any material, are the key constraints in gathering valid data. In general, crystals should be larger than 5-10 μm, which can be difficult to achieve for particular MOF classes. Infrared (IR) and Raman spectroscopy are the two basic types of vibrational spectroscopy. IR spectroscopy relies on absorbing infrared light, which induces a direct transition between the vibrational energy levels of molecules, whereas Raman spectroscopy relies on photon inelastic scattering. Since they are sensitive to various types of vibrations, the two strategies are complimentary. The infrared area is potentially split into three subregions. The fundamental vibrations are usually noticed in the mid-infrared (4000-400 cm^-1^). Overtones and combination modes are most commonly recognised in the near-infrared (14000-4000 cm^-1^). The low-energy far-infrared (ca. 400 to-10 cm^-1^) contains the terahertz region, which can be used to monitor intermolecular vibrations (external modes). In summary, IR is more responsive to hetero-nuclear functional groups (polar functional groups) than Raman is to homonuclear molecular bonds. Consequently, the IR and Raman spectra typically vary. Due to the differing basis sets, both methods (IR and Raman) are thought to be highly complementary [36]. Guest molecules immobilized on activated metal centres have been discovered to possess a wide range of vibrational frequencies and intensities, allowing IR spectroscopy to precisely ensure the presence of single metal sites in both amorphous and crystalline substances. Based on these, IR spectroscopy can provide useful and relevant insight into metal sites by analysing the adsorption performance of appropriate molecules such as CO, CO_2_, NO, and H_2_. CO, in particular, is a common probe due to its tiny size and sensitivity to reactivity with cationic or metallic sites [35].

## Drug loading strategies to MOF

MOFs exhibit the distinctive features of extensive surface area and structural divergence that simplify drug loading on the exterior or enclosed inside the pores by various loading strategies. Two types of drug-loading strategies are widely used, called the one-step and the two-step methods [37].

### One-step

In a one-step method, therapeutic actives are directly integrated with the MOFs during synthesis. This strategy employs uniform distribution and high drug-loading capacity. Nevertheless, controlling particle size, morphology, and physiochemical characteristics of prevailing MOFs is challenging. Additionally, it is crucial to take extra precautions to ensure that a drug is not damaged during the synthesis process [38].

#### One-pot method

The one-pot method includes the co-precipitation of the drug with the MOF amidst the synthesis. As a result, drug molecules are homogenously distributed inside the pores of MOFs [39]. It is a cost-effective technique for drug loading during the synthesis of MOFs. It speeds up the reaction process, minimises waste and gets beyond the MOFs' restrictively tiny porous structure [37]. Zeolitic imidazolate framework (ZIF) crystals have been demonstrated by Zheng *et al.* [40] to be capable of entrapping large drug and dye molecules. Molecule loadings can be adjusted and uniformly dispersed throughout the crystals. Additionally, scientists have shown that the one-pot method may be used to concurrently encapsulate target molecules and metal nanoparticles (NPs) inside ZIFs.

#### Co-crystallization

In laboratory research or industry, it is frequently used for drug loading. Drug and MOF co-crystallization could result in a 3D supramolecular structure incorporating the actives under minimal reaction conditions. Most notably, co-crystallization does not change the drug's physicochemical characteristics, which can be utilised to increase the drug's solubility and loading effectiveness [37]. Terekhova and colleagues [41] demonstrated that leflunomide could be loaded into γ-CD-MOF by impregnation and co-crystallization strategies, and the drug loading was equivalent to or greater than that achieved by an impregnation approach.

#### Drugs as organic linkers for MOFs

Drugs or their prodrugs could act as organic ligands to create MOFs and serve as MOF reservoirs by coordinating their respective functionalities with particular metal ions [37]. Vassaki and co-worker’s [42] reported phosphonate MOF-based drug delivery systems. They used a number of anti-osteoporosis bisphosphonate drugs (etidronate, pamidronate, alendronate, and neridronate) and bio-suitable metal ions (such as Ca^2+^and Mg^2+^) as the organic ligands.

### Two-step

The second method of drug loading involves encapsulating drugs within the structure of nMOFs that have already been manufactured in order to preserve particle form. Drugs are typically predicted to be entrapped into the scaffolds by hydrogen bonding, interaction, or other host-guest interaction when their molecular dimensions are less than the pore diameter of nMOFs. However, larger molecules of drugs with opposing charges are likely to be adsorbed by nMOFs by electrostatic interaction [38].

#### Impregnation

Due to their porous structure and accessibility to metal ions and tiny molecules, MOFs can be impregnated with precursors by a diffusion/deposition method. The process usually entails two steps: the MOF solids were submerged in the precursor-containing solution for impregnation, and the precursors that were adsorbed either became the final active species or underwent additional reactions (by reduction, decomposition or other chemical pathways) to create new functional phases inside the MOF matrices. MOFs offer a constrained environment to restrict the expansion of these useful species and prevent their aggregation. As a result, very stable MOFs are frequently needed since a series of reactions may be required before the synthesis of final MOF composites [43]. Devautour-Vinot *et al.* [44] performed caffeine encapsulation by the simple impregnation method in a series of UiO-66(Zr)-family MOFs.

#### Mechanochemical method

This method involves mechanically combining drugs and MOFs in a solid state, which is solvent-free, environmentally friendly, and cost-effective [37]. Nadizadeh *et al.* [45] synthesized, ibuprofen (IBU) -loaded- nMOFs {Cu2(1,4-bdc)2(dabco)}n and {Cu2(1,4-bdcNH_2_)2(dabco)}n (bdc=benzenedicarboxilic acid, and dabco=diazabicyclooctane) by ball-milling at room temperature in 2 hours. Noorian *et al.* [46] used a simple ball milling technique to load the model drugs 5-fluorouracil, caffeine, para-aminobenzoic acid, and benzocaine efficiently.

#### Covalent binding

The covalent binding method uses organic linkers and inorganic metal clusters to form covalent bonds in the MOF structure. Even though the attractive technique inserts different cargos into MOFs, there are frequently issues with the drugs slowly leaking out due to the very weak interaction force between the drugs and MOFs [37]. Morris *et al.* [47] developed the UiO-66-N_3_ (Zr_6_O_4_OH_4_(C_8_H_3_O_4_N_3_)_6_) nanostructures of a MOF. Using a strain-promoted click reaction, oligonucleotides were covalently functionalized onto the MOF's surface.

## Formulation development for MOF

As previously reported, controlling particle size is an important constraint in the field of biomedicine, particularly when taking into account how they are administered via the most popular routes (e.g., oral, intravenous, intranasal, cutaneous, ophthalmic, otic, and so on) [48]. The NPs' physicochemical characterristics, such as particle size, surface charge, rheological characteristics, and colloidal stability, will dictate how well they interact with various biological components and/or frameworks (formulations), how they are distributed in the body, and how effective they are. As a result, careful control over the particle size yields a necessary foundation for successfully implementing any form of NPs in biological applications [49]. In the case of nMOFs as nanocarriers for the delivery of therapeutics, recent studies have concentrated on morphology, particle size, and particle size distribution, which determine the final formulation according to the route of administration [48]. As previously mentioned, controlling particle size is the biggest task during the administration of the formulation via various routes, and it is also important to design a stable and reproducible formulation. When administering nMOFs *in vivo*, specific sets of criteria must be met in order to prevent embolism, including uniform particle sizes (˂200 nm) and highly stable water-based suspensions (free of aggregates or precipitation) [50]. In the case of optimisation of MOFs by the DoE approach, MOFs are promising candidates for DoE applications. By properly optimising synthesis parameters, including residence time, linker concentration, metal/linker volumetric ratio, and solvent selection, there are multiple orders of magnitude [51].

### Design of experiment

To develop an effective formulation of any drug, the screening and optimisation of components involved in it are extremely crucial. They also need to be optimized to identify and screen the various parameters that are likely to impact the final formulation. To optimise formulation, the most popular design of experiment (DoE) tools have been used. In this regard, understanding and optimising the variables that affect the production of NPs has been facilitated by focusing on quality manufacturing. DoE techniques have been used to this end to produce high-quality formulations by learning sufficient about the process [52]. DoE is a potent tool that particularly seeks to optimise and reduce the number of trials statistically, recognise the impact of the explored variables on the results, and anticipate the response of unproven circumstances within the range of the investigated experimental areas [51]. Regarding MOF formulation optimisation, the process of optimisation started with the synthesis of MOFs. Yingpeng Li and co-workers [53] designed the Hong Kong University of Science and Technology HKUST-1 for the efficient delivery of 5-FU. Researchers employed a central composite design to optimise the percent drug loading. They employed three independent variables for the optimisation: contact time, ethanol concentration and the 5-FU: material ratio. The effect of these variables is checked at three experimental levels, 1, 0, and -1, which are tested against response variables such as drug entrapment efficiency and percent drug loading. The designed three batches showed 40.23, 34.58, and 38.43 %, percent drug loading, respectively, with a standard deviation of 2.89 and p-value ˂ 0.05, proving that the optimized process was stable and reproducible. In a similar vein, P. Kush *et al.* [54] reported the optimisation and synthesis of MIL-101-NH_2_ by the microwave-assisted method. Further researchers and team used the central composite design for the optimisation, including three independent variables: reactant composition, pH and temperature. The resultant variables were observed in percent yield, size of MOF and surface area. The ANOVA test showed that the p-value ˂ 0.05, revealing a significant impact of selected independent variables on selected dependent responses.

### Types of drug delivery systems

During the last few decades, DDS has taken advantage of MOFs and novel frameworks [55]. Drug administration has always been a challenge for formulation scientists by virtue of the low aqueous or lipidic solubility, stability-related concerns, toxicity and limited biodistribution. But the advancement of MOFs in DDS has recognised that MOFs can address these issues [48]. Their amphiphilic internal environment, multifaceted composition, and adaptive porosity make them particularly appealing for hosting large numbers of guest moieties with varying physicochemical features. Along with the wide array of therapeutic compounds, the different types of active constituents can also be entrapped as a core part of the MOF [56]. In parallel, as it is possible to introduce a large variety of stimuli-responsive groups ranging from porphyrin, which is responsive to light, pH-responsive imidazolyl, *etc.*, groups into the MOF skeleton, the system can be designed to release drugs under stimuli of interest. In this part, we will get insights into different types of drug delivery systems, which can be either nonresponsive, single stimuli-responsive or multi-stimuli responsive [57].

#### Nonresponsive MOF

Nonresponsive DDS based on MOF does not show any response to external stimuli. In such systems, drug release can result from dissolution, diffusion or ion exchange mechanisms and with a change in the release mechanism, the drug release profile also changes. If the drug release from the MOF occurs owing to slow degradation or merely by diffusion, the drug release will be sustained for a longer time. On the other hand, if under any specific condition or in specific media, dissolution of MOF occurs, the resulting drug release will be a burst release. In general, for reducing toxic effects and getting steady blood concentrations, one might believe that a sustained release is preferable. Furthermore, the sustained release can protect the drug from undergoing any degradation as it increases its plasma half-life, resulting in better bioavailability and enhanced efficacy. Sustained release is not always preferred as it allows the drug to remain in the blood for a prolonged period. The release profile is solely dependent on the application, and accordingly, one should go for a selection of MOF. To tackle the problem of premature drug release, assorted preventive measures can be taken, which comprise coating lipid or silica layers and exosomes on the surface of MOF, which also results in improved solubility of MOF [58].

#### Stimuli-responsive MOF

**Figure 2.**
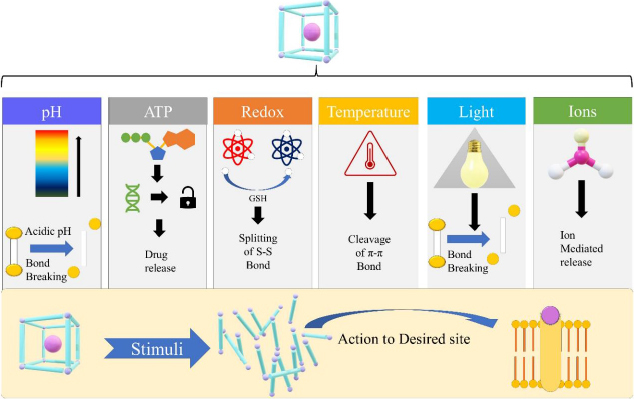
Different stimuli for stimuli-responsive MOFs

As mentioned earlier, MOFs are exceptional drug carriers because they have the least cytotoxicity, superior loading capacity, and compelling cell and tissue permeation. Even so, one of the dominant issues with MOF is the premature release of entrapped drugs. To tackle this problem, strategically designed MOFs responsive to external stimuli can be a satisfactory solution [59]. The MOFs are especially useful when the external stimuli are specific to disease pathology, allowing the MOFs to respond specifically to the pathological triggers. Generally, the stimuli can be categorized into external and internal groups. The internal stimuli include temperature, concentration of enzymes between diseased cells and normal cells, temperature, hypoxia, pH and redox potential. This type of stimulus, as illustrated in Figure 2, depends on the various physicochemical environments available at the site of action. On the contrary, external stimuli depend on external factors like light, ultrasound, heat, electric current and magnetic field. By this virtue, optimal spatio-temporal drug release in a controlled manner can be attained [9]. In the stimuli-responsive MOF, drug release can be attained by a single stimulus or by the combination of multiple stimuli.

#### Single stimuli responsive MOF

In this type of system, drug release can be attained by the application of single external or internal stimuli, which can be either pH, temperature, light, redox or ATP.

##### a. pH-responsive

Among the assorted classes of MOFs responsive to external stimuli, pH-responsive MOFs are one of the most studied, notably for cancer therapy. The change in the pH of the external environment may result in the pH-responsive breakdown of the MOF structure [57]. The release of the drug is due to the structural breakdown of MOF structures due to pH-dependent instability. In addition, MOFs responsive to pH have been widely investigated for the oral delivery of chemotherapeutics and anti-inflammatory agents [60]. In contrast to cancer therapy, in the case of oral delivery of anti-inflammatory agents in the acidified medium, the burst effect must be smaller in the acidic medium, *i.e.*, in the stomach and the drug release is mostly expected in intestinal conditions. The most preferable mechanism for the pH responsiveness of the MOFs is the utilization of the bond breaking by the protonation. When exposed to acidic circumstances, MOFs made with an organic ligand containing ionizable chemical groups are prone to be protonated for charge reversal; nevertheless, they stay deionized or deprotonated when pH is about 7.4. In another approach, the MOFs are attached to the therapeutic agent using covalent bonds, which results in a highly selective targeting response. This method involves hydrolysing acid labile bonds between MOF and therapeutic agent at low pH, which releases the therapeutics [61]. For the first time, Sun *et al.* [62] outlined a drug delivery system 5-FU@ZIF-8 (5-FU =5-fluorouracil). For this delivery system, the induced controlled drug release was obtained and the system showed a booming drug loading capacity of up to 60 %. The drug release experiments showed that in phosphate buffer saline (PBS), 5-FU demonstrated faster drug release at pH 5.0 than at pH 7.4. The percent drug release demonstrated that >45 % of drug release is obtained in the first hour at pH 5.0, while on the other hand, only 17 % was obtained at pH 7.4. In another work, Duan *et al.* [60] synthesized pH-responsive NPs based on MOFs for the delivery of drugs. This system was utilized for cancer immunotherapy, containing tumor-associated antigens (TAAs) and immunostimulatory unmethylated cytosine phosphate- guanine oligonucleotide (CpG). At pH 5.0, around 60 % antigen release was obtained and enhanced antitumor activity was exhibited when *in-vivo* studies were carried out on B16-OVA melanoma cancers. In the recent era, S. Guillen and his team [63] developed the pH-responsive thin film MIL-88 to analyse its potential against healthy and inflammatory or cancerous cells. The real-time observations of the designed film were made using surface plasmon resonance spectroscopy. The dissociation constant of the pH-responsive film was measured in an acidic microenvironment (pH 6.3), which was found to be 6.10 ± 0.86 ms^−1^, while in neutral pH conditions, it was ten times less in dissociation than in an acidic medium. The MIL-88 film was also examined by quartz crystal microbalance (QCM) to demonstrate IBU loading ability, which was nearly 6.0 μg cm^-2^. Moreover, the drug release profile of the film was performed in both acidic and neutral pH conditions (pH 6.3 and 7.4), respectively, to simulate the normal body and inflammatory or cancerous cell conditions. Over a span of 55 h of testing, it came to light that the amount of IBU released in acidic pH settings was much larger than that in neutral pH, *i.e.*, a healthy body system. In an acidic environment, the cleavage of bonds between the Fe^3+^ and carboxylate ligands triggers the release of IBU. The findings of the present study point out its potential in future applications as a pH-responsive MOF device. In 2023, Zi-Jian Zhang *et al.* [64] came up with a new approach for osteoarthritis (OA) therapy via the co-delivery of pH-responsive MIL-101-NH_2_ for co-delivery of curcumin (CCM) and small interfering RNA (siRNA) for hypoxia-inducible factor (HIF-2α). HIF-2α aggravates the hypoxia in OA, characterised by chronic, severe inflammation and irreversible cartilage loss and is strongly connected with decreased joint lubrication effectiveness. Still, pending concerns include the poor response to monotherapy approaches, unfavourable bioavailability-induced poor effectiveness, short retention, and inadequate stimulus reactivity. Herein, the si-RNA@CCM-loaded-MIL-101-NH_2_ system, designed by the encapsulation of CCM and si-RNA in MIL-101-NH_2_, has the ability to protect CCM and si-RNA by nuclease degradation from lysosomal escape. In acidic media, the MIL-101-NH_2_ started disintegrating, leading to the release of encapsulated CCM to minimise pro-inflammatory cytokinin’s level while releasing siRNA in order to act as gene-silencing therapy by cleaving the target HIF-2 mRNA. Eventually, demonstrating the effectiveness of therapy through the inhibition of OA's inflammation mechanism and cartilage deterioration by suppressing HIF-2 genes, owing to the findings of *in-vitro* and *in-vivo* studies, the pH-responsive MIL-101-NH_2_ has potential for OA therapy and may provide a platform for future OA therapies.

##### b. Light responsive

As the light-responsive approach has the upper hand in being eco-friendly, as well as serving some advantages such as spatial and temporal control over release, a non-invasive approach, easy fine-tuning, and operability, it has gained many researchers limelight for designing drug delivery applications [65]. All these applications considered, the light-responsive approach has been used to deliver the MOFs to attain spatiotemporal delivery. The prime mechanism involved in light-responsive delivery is the light-mediated chemical bond cleavage, reversible or irreversible conformational changes or photothermal transformation of molecules or groups [66]. Also, the ligand-based approach can be utilized for designing light-responsive MOFs by incorporating light-responsive molecules as ligands. For attaining light-responsive MOFs like UiO-ABZ and porphyrin MOFs, approaches like integrating photoactive moieties like indocyanine green (ICG), porphyrin, anthracene and its derivatives and, azobenzenedicarboxylate (AZB) into the MOF structure or by scheming reactive oxygen species are being utilized. These systems can generate singlet oxygen (^1^O_2_) in the presence of visible and NIR, which abolishes MOF structure to release drug molecules. This approach is collectively recognized as photodynamic therapy [66-68]. In line with this, Park *et al.* [69] utilized photodynamic therapy for targeted delivery by intending NIR light-induced reactive oxygen species (ROS) generating Zr(IV)-based porphyrinic MOF (Zr-MOF). These MOFs had the ability to accumulate at tumor sites through enhanced permeability and retention (EPR) effects upon injecting. While targeting ability is unsatisfactory, there may be an increased chance of side effects. To overcome this challenge, folic acid modification of Zr-MOF was done, which resulted in improved targeting and improved photodynamic therapy efficacy. In another case, Liu and co-workers [70] designed the double silica-shelled upconversion nanoparticles (UCNPs) nanostructure proficient at delivering bio-reductive tirapazamine (TPZ, a prodrug) and photosensitizer (PS) molecules. This system could achieve a synergistic effect first by UC-based photodynamic therapy under a standard oxygen environment and when oxygen is depleted by UC-PDT, the cytotoxicity of activated TPZ is then produced. In contrast to UC-PDT alone, treatment with TPZ-UC/PS and NIR laser resulted in noticeably decreased tumour growth, suggesting that the way TPZ is given has a significant impact on the effectiveness of treatments for the much-amended cytotoxicity of TPZ under PDT-induced hypoxia. In 2018, Dandan Xu [71] created the ZnPc@ZIF-8 with a one-step coprecipitation approach. ZnPc@ZIF-8 contains the water-insoluble photosensitizer zinc(II) phthalocyanine (ZnPc), a typical hydrophobic PS. ZIF-8's micropores serve as molecular cages that keep hydrophobic ZnPc monomeric and prevent it from self-aggregating. This allows the encapsulated ZnPc to produce lethal ^1^O_2_ in the presence of light irradiation (650 nm) in an aqueous environment. Cancer cells can endocytoze the ZnPc@ZIF-8 nano-system that has been created, and it exhibits red fluorescence emission with good photodynamic activity for cancer therapy *in vitro.* The self-quenching of ZnPc's fluorescence emission can also be used to monitor the acid sensitivity of ZnPc@ZIF-8, which would fully degenerate following PDT. The use of MOFs as nano-carriers in this research provides the path for an easy solution to the issue of the solubility and bioavailability of hydrophobic PS. In 2019, Aziza Sharsheeva and coworkers [72] proposed a study in which the research team combined drug delivery nanocarriers with a semiconductor photocatalytic component that could produce a local pH gradient in response to electromagnetic radiation from the outside. To put this theory to the test, researchers looked into a model drug-releasing nanocomposite made of ZIF-8, photocatalytic titania nanotubes, and the anticancer agent doxorubicin (DOX). Compared to the release duration in systems without a photocatalyst, which normally ranges from several hours to several days, this outcome was accomplished with locally applied UV irradiation in just 40 minutes, which was a comparatively very short period. The chances of survival of patients with malignant tumours could be significantly improved by immunotherapy testing utilising immunoadjuvants and tumour antigens. MOFs have been widely used in cancer therapy as efficient carriers because of their exceptional histocompatibility and minimal toxicity. Considering this, Zhijin Fan *et al.* [73] used a specific MOF (MIL101-NH_2_) as the core carrier to develop a multimodal imaging-guided synergistic cancer photoimmunotherapy. The MOF was dual-dressed in photoacoustic and fluorescent signal donors (indocyanine green, ICG), immune adjuvants (cytosine-phosphate-guanine sequence, CpG), and was given the name ICG-CpG@MOF. Through the EPR effect, this nanocarrier might passively target the tumour site and produce multimodal tumour imaging (fluorescent, photoacoustic, photothermal, and magnetic resonance imaging). Synergistic cancer photoimmunotherapy was accomplished through simultaneous photodynamic and photothermal approaches with 808 nm laser irradiation. Immunoadjuvant was successsfully released into the tumour microenvironment under GSH control thanks to ICG-CpG@MOF. Furthermore, the immune system might be stimulated by the secreted tumour-associated antigen and CpG to cause the transition of tumour cells from cold to hot. This dramatically increased tumour cytotoxicity and led to high cure rates with few side effects. A promising method for the detection and treatment of cancer is offered by this approach, which combines multimodal imaging and cancer photoimmunotherapy.

##### c. Thermoresponsive

The thermoresponsive MOFs have the ability to release the active moiety under the influence of a temperature change. In the case of thermoresponsive MOFs, the π-π interactions between host and guest get cleaved with a rise in temperature, causing the drug to get resealed [74]. From the class of numerous thermosensitive materials, poly(N-isopropylacrylamide) (PNIPAM) is recognized as a popular thermosresponsive material having a low critical solution temperature. This material gets easily dissolved in water below its cloud point, which is *T*c ≈31 °C, due to its hydrophilic nature while remaining undissolved above the cloud temperature [75]. With this background, Nagata *et al.* [76] utilized thermoresponsive polymer (PNIPAM) for the preparation of thermoresponsive MOFs (UiO-66-PNIPAM) by surface modification. Through conformational variations of PNIPAM implanted onto the MOF, this accomplished polymer-modified MOF nanocarrier demonstrated regulated release of the encapsulated guest molecules such as resorufin, caffeine, and procainamide with temperature variation. Drug-loaded UiO-66-PNIPAM showed fast drug release at a low temperature (25 °C). However, at a high temperature (40 °C), the drug release phenomena were suppressed above its *T*c (31 °C). On the other hand, the tumors have the ability to undergo localized heating, which results in an increased temperature of the nanocarriers within the tumors. In this regard, the MOF's structural changes brought on by the temperature shift result in cargo release. It is crucial for these systems that MOFs maintain their stability at 37 °C, which is the average body temperature [77]. In another study, by using zinc ions, the biomolecular linker adenine, and carboxylate-based ligands, two zinc-based porous MOFs, ZJU-64 and ZJU-64-CH3, sharing the same crystal structure, have been synthesised by Wenxin Lin [78] resulting in 3D frameworks with 1D channels (about 1.6 nm and 1.9 nm). The 3-(4,5-dimethylthiazol-2-yl)--2,5-diphenyl tetrazolium (MTT) assay, microscope images, and cell-cycle analysis of the materials ZJU-64 and ZJU-64-CH_3_ demonstrate low toxicity, indicating their potential applications for human health care. Methotrexate (MTX), an anticancer medication, may be successfully loaded into MOFs using a straightforward impregnation process. The drug payloads for ZJU-64 and ZJU-64-CH_3_ are approximately 13.45 and 10.63 wt.%, respectively. For several days, the sustained drug-release behaviour of MTX from MTX-loaded ZJU-64 and ZJU-64-CH_3_ is observed. The temperature-responsive drug delivery of ZJU-64 and ZJU-64-CH_3_ demonstrates that hyperthermia can regulate the drug release. It paves the way for a novel therapy that combines drug therapy and thermal therapy to kill more cancer cells while causing fewer side effects. This demonstrates the usefulness of ZJU-64 and ZJU-64-CH_3_ as "smart" drug carriers with greater therapeutic efficacy. In another study, the design, synthesis, characterization, and application of an adsorbent-heater-thermometer nanomaterial, (ZIF-8,EuxTby)@AuNP, based on ZIF-8 (adsorbent), incorporating Eu^3+^ and/or Tb^3+^ ions (thermometer), and gold nanoparticles (AuNPs, heater) was done by José Yago R. Silva [79]. These composite materials were identified as core-shell nanocrystals, with the crystalline ZIF-8 acting as the shell and the lanthanide ions being incorporated into or chemosorbed onto the AuNPs, which serve as the structure's core. The AuNPs serve as heaters, the ZIF-8 shell as a drug adsorbent, and the ligand and lanthanide ion luminescence intensities are employed to measure temperature. As demonstrated with the examples of 5-FU and caffeine, visible irradiation can be used to activate this thermoresponsive material and cause it to release tiny molecules under regulated conditions. It has been demonstrated through computer simulations and calculations using the transition state theory that the diffusion of tiny molecules between adjacent pores in ZIF-8 is extremely constrained and involves high-energy barriers. These results suggest that these molecules are uploaded onto and released from the ZIF-8 surface instead of being inside the holes. This is the first report of ZIF-8 nanocrystals (adsorbents) containing both heaters for regulated drug release in a physiological temperature range and lanthanide ions as sensitive nanothermometers. These findings offer a proof-of-concept that may be extended to other types of materials and a fresh viewpoint on the creation of multifunctional, self-assembling, thermoresponsive adsorbing materials that are quick to prepare and easy to regulate. In another study, Wei Wu and team [80] created soap-free emulsion polymerization, a core-shell nMOFs@poly(N-isopropylacrylamide) (PNIPAm) microgel hybrid through one-pot. By adjusting the concentration of the monomer and crosslinker in the reaction, the PNIPAm microgel layer on the surface of nMOFs can form in any pattern. By adjusting the temperature below and above the lower critical solution temperature, reversible swelling-collapsing behaviours of the hybrid are achieved. When employed as a water lubrication additive, the hybrid permits a decrease in friction coefficient and wear volume. *In vitro* thermal-responsive drug release is shown by manipulating the PNIPAm nanolayer's swelling and collapsing states on the diclofenac sodium-loaded hybrid. Additionally, the hybrid's strong biocompatibility is confirmed through the cultivation of HeLa and BEAS-2B cells. These findings build a nMOFs@microgel hybrid capable of reducing wear and friction while enabling thermally responsive drug release. In 2021, To increase the anticancer potential of norcantharidin (NCTD) and lower the dosage of the medication, Xiu-Yan Li [81] created a nanomaterial carrier, NCTD-loaded IRMOF-3 coated with a temperature-sensitive gel (NCTD-IRMOF-3-Gel). A coordination reaction produced NCTD-IRMOF-3-Gel. We looked into the apparent properties and *in-vitro* release of NCTD-IRMOF-3-Gel. The anti-liver cancer effectiveness of NCTD-IRMOF-3-Gel in *in-vitro* models was assessed using cell cytotoxicity tests, flow cytometry, and apoptosis investigations in mouse hepatoma (Hepa1-6) cells. NCTD-IRMOF-3-Gel had consistent particle size distribution and its particle size ranged from 50 to 100 nm. The release curve demonstrated the clear sustained-release impact of NCTD-IRMOF-3-Gel. According to the results of the cytotoxicity tests, the free drug NCTD and NCTD-IRMOF-3-Gel treatments significantly reduced the proliferative ability of Hepa1-6 cells, and the inhibition rate rose with increasing drug concentration. The Hepa1-6 cell cycle was found by flow cytometry to be blocked by NCTD-IRMOF-3-Gel in the S and G2/M phases, and the thermosensitive gel nanoparticles may prevent cell growth by causing cell cycle arrest. Studies on apoptosis demonstrated that NCTD-IRMOF-3-Gel caused Hepa1-6 cells to die. The outcomes suggested that the NCTD-IRMOF-3-Gel would be useful for treating liver cancer.

##### d. Redox responsive

When it comes to smart stimuli-responsive delivery systems, redox-responsive MOFs have become a topic of interest. This drug delivery system is based on the different redox concentrations in normal and tumor cells [82]. Redox-responsive frameworks have a sweeping influence on the delivery of antigens and other immunological areas. Glutathione (GSH) is present in much higher quantities in tumor cells than in normal body cells and acts as a potential reducing agent. The rapid splitting of the redox-sensitive S-S bond in the presence of GSH makes it an intriguing receptor location for the development of redox-responsive drug delivery systems. Considering this, Zhao *et al.* [83] synthesized a MOF system sensitive to GSH, which consists of Mn^2+^ ions and disulfide (S-S)-containing dithiodiglycolic acid as a ligand. This system (Mn-S-S) has the DOX loaded via hydrophobic interactions. Mn-S-S was coated with polydopamine (PDA) and further enhanced with polyethylene glycol (PEG) to create Mn-SS/DOX@PDA-PEG NCPs. For the effective release of the DOX encapsulated in the NCPs, the disulfide bond was severed in the presence of GSH. Magnetic resonance imaging (MRI) also revealed a higher T1 contrast for the Mn^2+^ in NCPs. The simple approach used for attaining redox-induced release is post-synthetic modification. This can be done by loading the MOFs with the active moieties and then blocking the pores with a disulphide-containing surface coating. The release occurs when MOFs come in contact with GSH present in cells, which causes the coating breakdown[84]. In this line, Ma and co-workers [85] used cystamine to coat ZIF-8 material for redox-induced paclitaxel (PTX) release. Researchers used cystamine as the linker and a redox-sensitive substance to create a redox-responsive PTX drug delivery platform based on MOFs. ZIF-8 served as the drug delivery vehicle. TEM was used to analyse the morphology of ZIF-8, and XRD was used to analyse the crystal structure. The surface modification of ZIF-8 was investigated using FT-IR spectroscopy. According to the BET analysis, surface modification had no effect on ZIF-8's specific surface area and pore size distribution. Under various pH and GSH concentrations, the drug release of ZIF-8, cystamine, and PTX was investigated. In a different work, Bingqian Lei [86] used iron, aluminium, or zirconium as the metal nodes and 4,4′-dithiobisbenzoic acid (4,4′-DTBA) as the organic ligand to construct a novel intrinsic redox-responsive MOFs carrier, MOFM(DTBA) (M = Fe, Al or Zr). It is possible for GSH, frequently overexpressed in tumour cells, to break the disulfide link in 4,4′-DTBA. It was discovered that MOF-Zr(DTBA) produced at 40 °C has the right shape and characteristics for a drug carrier. CCM was added to MOF-Zr(DTBA) to create CCM@MOF-Zr(DTBA) nanoparticles, which released more quickly *in-vitro* and caused more cell death than free CCM. According to *in-vivo* anticancer tests, CCM@ MOF-Zr(DTBA) has much better antitumor activity than free CCM. This method for creating responsive MOF-based nanocarriers may present fresh opportunities for using MOFs in theranostics, molecular imaging, and drug delivery. In a similar line, Haozhe He [87] demonstrated that a hybrid PFP@Fe/Cu-SS MOF increased intratumoral LPO content by redox reactions producing OH. LPO level also increased as a result of the inactivation of glutathione peroxide 4 (GPX4) caused by the depletion of GSH through disulfide-thiol exchange. This MOF significantly slowed their growth when Huh-7 tumours were xenografted onto mice. When a ferroptosis inhibitor is also administered, the MOF's anticancer action is lessened, which results in a recovery of GPX4 activity and an acceleration of tumour growth. Additionally, by including Cu in mesoporous PFP@Fe/Cu-SS, the MOF was able to retain its photothermal conversion capability while simultaneously serving as a contrast agent for T1-weighted magnetic resonance imaging. As a result, near-infrared light caused photothermal treatment and turned the liquid perfluoropentane enclosed into microbubbles for ultrasound imaging.

##### e. ATP responsive

Adenosine triphosphate (ATP), a high-energy unstable compound, is widely present throughout all living organisms. The mechanism for ATP responsiveness can be classified into two groups: 1) ATP-aptamer complex formation and 2) ATP-ion complex formation. In the first case, a stable ATP-binding DNA structure is formed by binding DNA to the agarose column with two piled G-quartets. The two conserved adenosine residues can stack between the top G-quartets and the short stems, forming a pocket structure where the adenosine or ATP ligand can attach. It is demonstrated that the synthesis of nucleic acid hairpins or the complexation of sDNA with complementary DNA might lock cargos in the pores of MOFs [88]. In this regard, Chen and co-workers used an nMOF for drug delivery that contained Zr^4+^ ions, amino-triphenyldicarboxylic acid, and a complementary nucleic acid (the ATP aptamer sequence or the ATP-AS1411 hybrid aptamer sequence). The release of drugs is followed by the unlocking of these frameworks in the ATP environment by the formation of an ATP-aptamer complex. Only 10 % of MCF-10A epithelial breast cells perished under comparable circumstances when DOX-loaded ATP aptamer-gated or ATP AS1411 aptamer-gated nMOFs were used to treat MDA-MB-231 breast cancer cells. The targeting and efficient penetration of the ATP-responsive nMOFs into the cancer cells increased the cytotoxicity because ATP is overexpressed in cancer cells and the AS1411 aptamer recognises the nucleolin receptor sites on their cell membranes [89]. Another facet for targeting breast cancer through the ATP-responsive coating of nucleic acid-based polyacrylamide hydrogel @DOX-loaded-MOF was introduced by Wei-Hai Chen and colleagues [90]. The linkage of a pair of polyacrylamide chains, PA and PB, which are modified with two nucleic acid hairpins (4 made up of 5′-ACATCCCCTTCCTCCGAGCTGACCTGGGGGAGTATTGCGGAGGAAGGTACCAGATCTAGAGC-3′; sequences of DNA) and (5 made up of 5′-Acryd/TTTTTTTTCCTGGGGGAGTATTGCGGAGGAAGGGGATGTCTCCCCCAGGTCAGCT-3′ sequences of DNA) utilising the strand-induced hybridization chain reaction, promotes the production of the hydrogel. The generated anti-ATP aptamer repeat is enclosed in the polyacrylamide gel with duplex bridges. The hydrogel shell secures the drugs contained in the nMOFs. The hydrogel layer is broken down by creating the ATP-aptamer complex in the vicinity of ATP, which is elevated in cancerous cells. This resulted in the release of the DOX. The modified nMOFs address important shortcomings of the previously outlined nucleic acid-gated drug-loaded nMOFs in lieu of providing an umbrella approach for the synthesis of drug-loaded ATP-responsive polyacrylamide hydrogel-coated nMOFs. The research clarifies that the hydrogel-coated nMOFs have substantially greater drug loading than the nucleic acid-gated nMOFs, preventing off-target drug leakage reported with the nucleic acid-secured nMOFs. In comparison with healthy MCF-10A breast cells, the dox-encapsulated, ATP-responsive, hydrogel-coated nMOFs exhibit specific and potent cytotoxicity towards MDA-MB-231 breast cancer cells. In a similar vein, Xiaoti Yang *et al.* [91] reported the ZIF-90 nanoplatform for the delivery of cytosolic protein along with clustered regularly interspaced short palindromic repeats (CRISPR/Cas-9) genome editing. Imidazole-2-carboxaldehyde, Zn^2+^ and protein self-assemble to generate ZIF-90/protein nanoparticles, effectively enclosing protein. It was discovered that the competitive coupling between ATP and the Zn^2+^ of ZIF-90 causes the degradation of ZIF-90 NPs in the vicinity of ATP to liberate protein. According to intracellular delivery research, the ZIF-90 NPs are capable of delivering a wide range of proteins inside the cytosol, irrespective of protein size and molecular mass. As cytotoxic RNase A is effectively delivered, tumour cell development is inhibited, and while the genome-editing protein Cas9 is successfully delivered, HeLa cells' expression of the green fluorescent protein (GFP) has been wiped out with an effectiveness of up to 35 %. The levels of ATP are elevated in disease cells, so it is anticipated that the administration of an ATP-responsive protein would create new possibilities for enhanced protein delivery and CRISPR/Cas9 genome editing to treat target-specific ailments.

#### Multi-stimuli responsive MOF

##### a. pH and temperature-responsive

Cancerous cells have certain distinct features, such as a lower pH and higher temperature. These features have opened the doors for new ways of controlling and treating cancer growth. Owing to the pH and temperature features, new gates have benefited by designing a dual pH and temperature-responsive system. Certain types of amino acids have a tendency to act as a pH-responsive ligand. In 2018, Lin and his colleagues [92] created the biocompatible MOF (Zn-GA) by combining L-glutamic acid with zinc ions. The MOFs were created by reacting L-glutamic acid with Zn(NO_3_)_2_x6H_2_O and adding it to DMF/H_2_O. The resulting Zn-GA has been separated and MTX-loaded after being heated to 80 °C. The nanocomposite demonstrated a suitable loading capacity of 12.85 wt.% and the ability to release drugs in response to heat and pH stimuli. Notably, at an acidic pH of 5.0, only 43 % of the medication was released in a normal physiological fluid; however, when the temperature rose, the drug release rate climbed to 68 %. The drug-free nanocarriers demonstrated high biocompatibility, whereas the PC12 cell viability was drastically reduced to 12 % following drug loading, demonstrating their anticancer potential. The outcomes confirmed the possibility of using nanocarriers for cancer treatment. A study by Li-Li Tan [93] provided a novel method for creating "gated scaffolds" that are sensitive to multiple stimuli, combining capped MOFs with supramolecular pseudorotaxanes. These mechanical Zr-MOFs demonstrated minimal cytotoxicity, great drug encapsulation, minor premature release, and good biocompatibility. The pH, lysosomal pH, and osteoclast pH are seen to be decreased around or inside the bone tumour cells (acidosis), and the ensuing osteolysis raises the Ca^2+^ concentration (hypercalcemia). The simultaneous changes in pH and Ca^2+^ content in bone cancer cells caused the mechanised MOFs to release their drugs. As a well-liked method of cancer treatment, hyperthermia, also known as thermal therapy or thermotherapy, can regulate medication release in the aforementioned system. The development of intelligent biomaterials for cancer treatment and bone regeneration is now possible because of this design. In another study, Shunjiro Nagata [94] showed that an MOF may release a guest molecule (procainamide) under regulated conditions. Researchers coated the MOF in post-synthetic modification coats with a copolymer of N-isopropyl acrylamide (NIPAM) and acrylic acid (AA). The hypothesis was that the guest molecule may be released from the MOF in an "on-off" fashion thanks to the polymer's quick and reversible coil-globule transition, which is pH- and thermosresponsive. The release of the guest molecule from the MOF is repressed at low pH (4.01) and high temperature (> 40 °C), where it was rapidly released at high pH (6.86) and low temperature (25 °C), respectively, when the polymer adopts a coil conformation. Even once the release has begun, it can be stopped by adding external stimulation. Targeted medication distribution and the regulated release of therapeutic substances will be made easier by the controllable container generated from MOF. A multifunctional nanoplatforms designed to offer PDT, PTT, and CT was described by Yao *et al.* [95]. Initially, a dispersion of zirconium tetrachloride in DMF, 2-aminoterephthalic acid, benzoic acid, and hydrochloric acid was heated at 120 °C for 48 hours to create the zirconium framework (UiO-66-N_3_). In order for the framework to function as a photosensitizer, it has been conjugated with a Ru(II) polypyridyl alkyne group. Additionally, DOX has been loaded as a prototype chemotherapy drug. After that, CuS nanoparticles were physically adsorbed onto the nanoplatform's surface to serve as a PTT agent (UiO-Ra-DOX-CuS). Here, the conjugate's ideal drug loading capacity (13.5 %) has been observed. It's interesting to note that UiO-Ra-DOX-CuS successfully produced 1O_2_, ensuring its potential in PDT. Notably, the temperature has linearly increased as the concentration of nanoparticles has continuously increased, pointing to a photothermal action. Approximately 30.5 % of DOX was released at physiological pH, whereas 48.5 % of the medication was released at pH 5. Importantly, after NIR irradiation, drug release was significantly encouraged. The UiO-Ra-DOX-CuS treatment had a greater synergistic impact (almost 100 % of the cells were eliminated) compared to monotherapy CT-42 % and laser irradiation (59 %), according to the cell line investigation on MDA-MB-231.

##### b. pH and magnetic-responsive

Under the influence of alternating magnetic fields, magnetic materials tend to generate heat through hysteresis losses caused by magnetic fields. This phenomenon can be applied to magnetic hyperthermia therapy (MHT). Magnetic stimuli MOFs are the potential candidates for improving the therapeutic efficacy of MHT. Recently, to understand the synergistic activity of MHT along with chemotherapy, the Fe3O4@PDA@ZIF-90 framework has been synthesized. The pH-dependent release profile has been studied as the system showed the highest release of 88.7 % of DOX at pH 4.5[96]. A lot of consideration has been given to the Cu-BTC framework in recent years as a potential drug carrier for cancer treatment because of its distinctive structural characteristics and prospective biocompatibility. A magnetic nano/microscale MOF has been successfully fabricated by Zahra Gharehdagh and coworkers [97] by incorporating Fe_3_O_4_ nanoparticles as an imaging agent into the porous isoreticular MOF [Cu^3^(BTC)_2_] as a drug carrier. However, this material's inherent incapacity for medical imaging may limit its bio applications. The synthetic magnetic MOFs display an outstanding pH-responsive drug release and a high loading capacity (40.5 %) towards the model anticancer DOX. The suggested nanocomposite not only has a lot of surface area, a lot of magnetic response, a lot of mesopore volume, a lot of transverse relaxivity (r2), and good stability, but it also has a lot of tumor-specific cellular uptake and significantly inhibits cancer cell viability without the use of any targeting agents. The synthesised magnetic nano/microcomposite is anticipated to be useful in medicine and to operate as a platform for pH/GSH/photo-triple-responsive nanocarriers and photoactive antibacterial treatment. In another study, Rezvan Shahin *et al.* [98] designed a nanocomposite, Fe_3_O_4_@UiO-66-NH_2_@PEwith pH-responsive and magnetic responsive ability for drug delivery. After being artificially modified via the Schiff base reaction between the amino groups in UiO-66-NH_2_ and the aldehyde groups in glutaraldehyde, a functional MOF based on zirconium was created: Fe_3_O_4_@UiO-66-NH_2_. Analysis techniques such as FT-IR, XRD, VSM, TGA, and SEM were used to find the synthesised carriers. The encapsulation effectiveness of core-shell nanocomposites was determined using UV spectrophotometry and obtained at 94 % due to the drug's non-volatile nature. Additionally, the drug load efficiency findings and the MTT technique, which revealed little cytotoxicity of nanomaterials even after that, are in excellent agreement. The resultant nanomaterials may contain any tiny biological molecule inside their channels and porosity, stabilise the environment, and do so with minimal cytotoxicity thanks to polymer coatings. However, the polymers gradually broke down and constantly released the drug after being exposed to various buffers and time. Furthermore, the nanocomposites exhibited pH-sensitive behaviour. Last but not least, the MTT test demonstrated efficient cytotoxicity, a positive finding for future biological applications [98]. By grafting -cyclodextrin onto the surface of Fe_3_O_4_@silica@MIL-100(Fe), Aseman Lajevardi and his colleagues [99] created a new magnetic and pH-responsive porous nanocomposite. Cephalexin's adsorption on the produced nanocomposite and its release behaviour were examined in relation to pH and temperature. The highest adsorption was discovered to occur at ambient temperature, whereas quick release occurred at higher temperatures. According to the data, the Langmuir isotherm model outperformed other two-parameter isotherm models in terms of accurately describing the drug's adsorption. To examine the mechanism of adsorption, a variety of kinetic models, including film transfer, intra-particle diffusion, and pseudo-second-order kinetic models, were utilised. Thermodynamic analyses showed the spontaneous and exothermic adsorption of cephalexin onto the nanocomposite. The nanocomposite further exhibited sensitive pH-dependent behaviour. The kinetics of release were well captured by the Higuchi model, which was researched to explore the drug release profile. Other mathematical kinetic models studied were zero-order, first-order, Korsmeyer-Peppas, and Higuchi.

##### c. Thermal and ion-responsive

Zn^2+^ has a crucial role in the different organs of the body, especially the nervous system. By considering this, the researcher has investigated Zn^2+^-responsive MOFs that respond to the Zn^2+^ imbalance in neurons and can effectively treat nervous disorders. Tan *et al.* used quaternary ammonium salt (Q) to modify UiO-66-NH_2_ and cap it with CP5 to assess the release of 5-FU under elevated temperature and increased Zn^2+^ concentrations. When Zn^2+^ builds up in the medium, the Zn^2+^ions have a tendency to bind to CP5 and open the drug-loaded pores. In this investigation, 5-FU released less than 25 % in Zn^2+^ free medium, but when Zn^2+^ concentration grew, 5-FU release increased to 75 % in 10 mM Zn^2+^ containing media. Also, as the release of 5-FU at 60 °C has risen to approximately 70 %, the interaction between CP5 and Q may be sensitive to temperature rise [100].

##### Routes of administration for MOF

**Figure 3.**
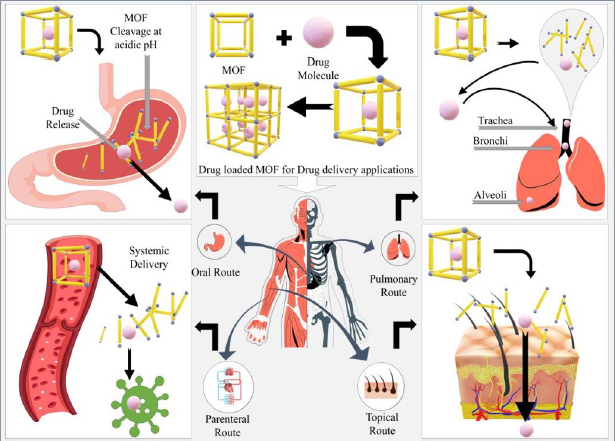
Routes of MOF administration

The drug delivery applications of MOFs have greatly drawn out in recent years as these systems can deliver therapeutics with high precision and loading due to their large surface area. Conventionally, only small molecular therapeutics were thought of as being administered via MOF delivery, but now, a large variety of macromolecular therapeutics like nucleic acids and proteins are being administered via MOFs. In this part of the review, we discuss the various routes of administering MOFs, as portrayed in Figure 3, for selective and non-selective delivery.

##### a. Oral

Nanosized biocompatible MOFs have always been significantly premeditated for drug delivery applications through different routes of administration but used less extensively than conventional oral routes of administration as, until now, the main focus was on increasing the bioavailability of the loaded drug without taking into account the intestinal permeability. In comparison with Fe(iron) -based nano MOF systems, newly synthesized MLL-127 has a better permeation through the intestine. Further intestinal permeability was improved by surface modification through chitosan coating. The intestinal permeability and biocompatibility were confirmed by the *Caenorhabditis elegans* in vivo model. *Caenorhabditis elegans* are worms that can ingest a large number of nano MOFs. At last, the ex vivo rat intestinal model confirmed the system's passage across the intestinal barrier for only 2 hours. The study showed that MOFs can act as a potential system for oral administration and improve intestinal permeability [101]. Previously, a similar study was carried out by Yuhao Zhou and his research team [102]. The team developed a nanocomposite-based MOF system for the oral delivery of insulin to tackle problems of low intestinal permeability and gastric degradation. They synthesized a nanocomposite biodegradable microsphere incorporating MOF NPs to overcome these barriers. A Fe-based MOF was fabricated first as a carrier with a loading capacity of 35 % and these were modified with sodium dodecyl sulfate for improving permeation across the Caco-2 monolayer *in-vitro* models. Further, these NPs were incorporated into biodegradable microspheres to protect the system from gastric degradation, which showed better stability in acidic media while releasing insulin in simulated intestinal fluid. *In-vitro* studies performed on BLAB/c nude mice showed that following oral administration, this system showed improved insulin availability compared to free oral insulin administration and showed improved plasma insulin levels for more than 6 h in diabetic rats, proving that these insulin containing MOF loaded microspheres can act as an effective means of oral delivery. In 2018, Siamak Javanbakht [103] used Cu-MOF porous blocks to be immersed in a pharmacological solution while loading IBU into two-dimensionally shaped tunnels and empty face-centered cubic cubes. The pH-sensitive biopolymeric gelatin microspheres were used to preserve the Cu-based MOFs-IBU Nanohybrid (Cu-MOF/IBU). According to the results, the manufactured gelatin microsphere may serve as a suggested capsule for the medication under gastrointestinal tract conditions. Utilizing FT-IR, XRD, UV-Vis, and SEM analyses, the gelatin-encapsulated Cu-MOF/IBU microsphere (Cu-MOF/IBU@GM) was characterised. *In vitro* drug delivery tests were conducted to demonstrate the effectiveness of the innovative microsphere as a controlled drug delivery device. The MTT assay was used to determine the cytotoxicity of the Cu-MOF, Cu-MOF/IBU, and Cu-MOF/IBU@GM compounds. Cu-MOF/IBU@GM showed very little cytotoxicity to Caco-2 cells after being incubated at various periods and concentrations, and the cell viability remained over 60 %. In contrast, Cu-MOF and IBU/Cu-MOF showed substantial cytotoxicity to Caco-2 cells even at low doses. Drug release studies showed that Cu-MOF decomposition in an acidic solution caused fast drug release at pH 1.2 (95 % of the drug released at 2 h) for Cu-MOF/IBU while Cu-MOF/IBU@GM released the drug in a controlled manner (pH 1.2:pH 6.8:pH 7.4-3:3:2) that may be related to the gelatin polymer's pH sensitivity and the polymeric network's diffusion barrier. A greater defense against drug release at low pH and a controlled release in alkaline conditions are demonstrated by primary drug release tests of IBU liberation from the Cu-MOF/IBU@GM processes. The results summarize that the system can be effectively used for oral delivery. On the other hand, although oral peptide or protein delivery is regarded as a ground-breaking substitute for daily subcutaneous injections, significant obstacles still exist in the stomach environment and the intestinal epithelium, which is obstructed by mucus and the epithelial cell layer and results in low bioavailability. Yuhao Zhou and team [104] created a pH-triggered self-unpacking capsule containing zwitterionic hydrogel-MOF NPs to prevent gastrointestinal degradation and encourage penetration across the intestinal mucosa. The zwitterionic hydrogel layer gives the nano-vehicles a special ability to penetrate past the mucus layer and effective internalization by epithelial cells. The MOF nanoparticles have a high exendin-4 loading capacity. Along with their gastro-resistance, pH-responsive capsules also drastically dissociate in the intestinal environment due to the quick formation of numerous CO_2_ bubbles, which leads to the abrupt release of the nanoparticles. In a diabetes rat model, orally administering the capsules containing exendin-4-loaded nanoparticles results in markedly elevated plasma exendin-4 levels for over 8 hours, which triggers noticeably boosted endogenous insulin release and an outstanding hypoglycemic effect with a relative pharmacological availability of 17.3 %. This oral exendin-4 method will offer significant potential for everyday and simple diabetic therapy due to the reduced risk of hypoglycemia. In 2022, Meng Gao and coworkers [105] developed a hybrid hydrogel-MOF system for the oral delivery of siRNA for the management of ulcerative colitis. Small interfering RNAs can be effective means of treatment for various inflammation-related disorders. Even though the gastric degradation of siRNA limits its use in cases of ulcerative colitis. To overcome this, the research team has attempted to load siRNA in MOF followed by sodium alginate encapsulation (SA@MOF-siRNATNFα) to promote oral absorption and stability. These hybrid platforms not only withstand low gastric and intestinal pH but also show improved intake by the inflammatory macrophages, resulting in a better release of SA@MOF-siRNATNFα. This results in better delivery of SA@MOF-siRNATNFα into local colon tissues and reduces the progression of colitis, which was confirmed by the in vivo mice model and treated mice do not exhibit any signs of weight loss, diarrhea or bloody stools. A conclusive study has long-established that designing hydrogel-MOFs hybrids can act as an effective means of delivery of therapeutics that tend to degrade in gastric media and can be a potential means of the oral delivery system for the delivery of siRNA in ulcerative colitis. As in another case, colon cancer is the fourth most prevalent type of cancer overall. Designing effective oral delivery systems received increasing focus in the development of novel controlled-release approaches. To do this, Siamak Javanbakht *et al.* [106] encapsulated 5-FU within the porous MOF-5 made of Zn. The 5-FU encapsulated MOF-5 nano-hybrid (5-FU@MOF-5) was protected and transported through the digestive system using a pH-sensitive carboxymethylcellulose (CMC) biopolymer. Different methods were used to characterize the CMC-coated 5-FU@MOF-5 bio-nanocomposite hydrogel bead (CMC/5-FU@MOF-5). FTIR studies proved that the FT-IR spectrum of 5-FU@MOF-5 is a mixture of the FT-IR spectrum of MOF-5 and 5-FU and has the characteristic bands associated with the MOF-5 and 5-FU. It was discovered that there was a rise in the intensity of a smaller band in the 400-800 cm^1^ range associated with the Fe-O band by comparing the FT-IR spectra of pure CMC and CMC/5-FU@MOF-5 beads. Additionally, after the production of the CMC/5-FU@MOF-5 hydrogel bead, the distinctive CMC bands at 1322 cm^1^ were reduced. The CMC/5-FU@MOF-5 hydrogel bead's cross-linked CMC polymeric chains with Fe^3+^ successfully, according to the FT-IR data. For MOF-5 and 5-FU@MOF-5, the BET surface area measurements yielded 147.8 and 106.4 m^2^ g^-1^, respectively. The MOF-5 showed an entrapment efficiency of 84.1 %. To determine the potential of the synthesised carrier as an oral 5-FU delivery method, drug release studies were conducted in a simulated GIT. All medicines were released from the 5-FU@MOF-5 over 3.5 hours due to MOF-5's breakdown at an acidic pH [65]. Contrarily, in CMC/5- FU@MOF-5, the amount of medication released is 70 % at 8 hours in a regulated way (pH 1.2: pH 6.8: pH 7.4-2:4:1), which is connected to the pH-sensitive nature of CMC biopolymer and the diffusion barrier of the hydrogel beads. The 5-FU@MOF-5 could be protected from potential degradation by CMC with shrinking at acidic pH, and CMC with time-dependent swelling at pH 6.8 and 7.4 demonstrated sustained release of 5-FU. Overall, the produced bio-nanocomposite hydrogel beads could be suggested as a possible drug delivery device for the colonic administration of 5-FU in light of the results obtained. In a different study, Ke Jiang and colleagues [107] created and synthesised ZJU-64-NSN, a novel anionic MOF having 1D channels adorned with highly polarized thiadiazole groups. Using a straightforward and environmentally friendly method, this MOF's crystal size could be systematically adjusted from 200 to 300 nm. This leads to the discovery of the ideal nanosized ZJU-64-NSN, which enables very quick loading of the cationic API procainamide (PA) (21.2 wt.% in 1 min). Moreover, the surface coating of polyethylene glycol (PEG) biopolymer significantly increases the unfavorable chemical stability of PA@ZJU-64- NSN. Due to the intensive host-guest interaction, the final drug delivery system PEG/PA@ZJU-64-NSN successfully blocks PA from premature release under tough stomach conditions and primarily releases PA to the targeted intestinal surroundings. The study on the dynamic behaviour of drug release and UV-Vis absorption spectrum clearly demonstrates that such controlled drug delivery is caused by endogenic Na ions rather than H ions. Using the MTT assay and confocal microscope imaging, the good biocompatibility of ZJU-64-NSN and PEG-coated ZJU-64-NSN was demonstrated. In a different study, Jun-Jie Zou [108] created highly effective oral insulin administration using acid-resistant MOF NPs (UiO-68-NH_2_) that were coated with targeting proteins (transferrin). High insulin loading was made possible by the UiO-68-NH_2_ nanocarrier with the right pore size, which also prevented acid and enzymatic destruction of the insulin. The transferrin-coated nanoparticles achieved effective transport across the intestinal epithelium and controlled insulin release under physiological settings, resulting in a substantial hypoglycemic impact and a high oral bioavailability of 29.6 %. This was accomplished by receptor-mediated transcellular route. The research showed that oral biomacromolecule distribution is possible because of the ability of functional MOFs to shield proteins from the gastric environment and cross the intestinal barrier.

##### b. Parenteral

Since the discovery of MOFs and their use in drug delivery applications, the parenteral route, which might involve the intravenous route, intramuscular route, subcutaneous route and many more, is a convenient route for the rapid delivery of cargo into the systemic circulation. In this regard, Gong Cheng and his team [109] developed a biomimetic platform based on MOFs for the intracellular and systemic delivery of functional therapeutic proteins and biofunctional enzymes. The MOFs showed a high loading capacity of 94 % of proteins, 50 times greater than surface conjugation. Further surface modification with extracellular vesicle membrane was done. The *in vitro* and *in vivo* studies proved that the MOFs protected the proteins against degradation by protease enzymes and bypassed immune system clearance. Furthermore, the system targeted homotypic tumor sites, promoting the uptake of MOFs by tumor and burst release inside the tumor cells and inhibiting tumor growth and around 14-fold improving therapeutic activity. Conclusively, this work demonstrated that MOFs can be a potential platform for intracellular and systemic delivery. In another study, Wenting Shang and team [110] demonstrated a MOF that, after labelling the targeted peptide iRGD, a theranostic nanoplatform would combine medication treatment with phototherapy. The micropore Fe-MOF was employed as a nanocarrier to upload chemotherapy medicines and MRI agents for identifying tumours. Additionally, under various delivery methods, including intravenous injection for breast cancer and local instillation for bladder cancer, MOF demonstrated outstanding targeting efficacy. In particular, the agent exhibits excellent photothermal treatment effectiveness and medication heat release efficiency when exposed to an 808 nm laser. The agent shows the great efficacy of photothermal therapy and the effectiveness of the medicine in releasing heat from the tumour location. This combination therapy offers a different way to administer medications and can be tailored to a variety of cancer cell types and molecular targets linked to the development of the disease. In a similar vein, Khaled Abou and colleagues [111] developed two nanoformulations consisting of two MOFs, Ti-MOF and Zr-MOF, both having surface changes, and each one filled with rutin (Rut) and/or piperine (Pip). *In vivo* anti-inflammatory and antioxidant properties were tested on MOFs and nanoformulations. It was possible to produce loadings of 27 wt.% for dual loading and 17 wt.% for a single pro-drug. For the first 12-hour stage, the release patterns for Rut and/or Pip followed a zero-order, and for the second stage, a stable, sustained release. The zero-order and Korsmeyer-Peppas kinetic models best fit the release patterns at pH 7.4. The anti-inflammatory and antioxidant effects of the nanoformulations were greater than those of either of their constituents, and those containing Rut or Pip alone had more potent effects than those including both agents. Leukocyte migration, paw edema model, and plasma antioxidant capacity assay results were consistent. The results showed that these drugs' anti-inflammatory and antioxidant properties are enhanced in nanoformulations compared to free form. Cheng Zhang [112] developed a new glucose-responsive delivery system (ZIF@Ins&GOx) for self-regulated insulin release by encapsulating glucose oxidase (GOx) and insulin in pH-sensitive ZIF-8 nanocrystals. When glucose enters the cavities of ZIF-8, GOx can convert it into gluconic acid, lowering the area's pH. The degradation of ZIF-8 nanocrystals in the presence of an acidic microenvironment would thus result in insulin release in a glucose-responsive manner. Studies conducted *in vitro* suggested that ZIF-8's stiff structure might shield insulin's biological action and that insulin release might be adjusted in response to glucose levels. In vivo tests showed that type 1 diabetes (T1D) patients could stabilize their blood glucose level at a normoglycemic state for up to 72 hours after receiving a single subcutaneous injection of the ZIF@Ins&GOx. A new proof-of-concept for the treatment of T1D is demonstrated by the multifunctional insulin delivery system using ZIF-8 nanocrystals loaded with an enzyme and insulin. Michael A. Luzuriaga [113] published a thorough analysis of ZIF-8 and its capacity to shield a model viral vector from denaturing circumstances. Combined spectroscopy and immunoassay analysis showed the improved thermal and chemical stability conformational makeup of the enclosed viral nanoparticle. The virus-ZIF composite's long-term biological activity was explored in animal models to clarify the consistency of the biosafety, immunogenicity, and encapsulated virus of the composite overall. Furthermore, histological examination of mice's skin and essential organs revealed no discernible tissue damage after numerous subcutaneous injections. This study demonstrates that ZIF-based protein composites are excellent candidates for better proteinaceous drug storage, are biocompatible and can regulate drug release and adsorption *in vivo*. In 2021, Mengyun He and team [114] proposed a type of "core-shell"-structured glucose-responsive nanoplatform for intravenous "smart" insulin delivery. The "inner core" was created by co-encapsulating insulin, GOx, and catalase (CAT) into ZIF-8 nanoparticles using a finely controlled one-pot biomimetic mineralization technique. An effective enzyme cascade system (GOx/CAT group) served as an optimised glucose-responsive module that could quickly catalyse glucose to yield gluconic acid to lower the local pH and effectively consume the harmful byproduct hydrogen peroxide. The "outer shell" of the "inner core" was covered with the erythrocyte membrane, a type of naturally occurring biologically derived lipid bilayer membrane with intrinsic biocompatibility, making the NPs injectable intravenously and able to sustain a long-term existence in blood circulation. The *in vitro* and *in vivo* results show that our expertly designed nanoplatform has excellent glucose-responsive properties and can keep type 1 diabetic mice's blood glucose levels at the normoglycemic state for up to 24 hours after being administered intravenously. This supports the use of an intravenous insulin delivery strategy to make up for the shortcomings of daily multiple subcutaneous insulin administration and provides a promising alternative.

##### c. Others

Even though nano MOFs are potential drug carriers, different formulations are the least explored, and most of their formulations are limited to only intravenous routes. On the other hand, lungs have a vast surface area, the least chances of enzymatic degradation, and can target both local and systemic delivery, but still delivering MOFs by pulmonary route is a less explored area. Cristina Fernández-Paz and team [115] engineered nano MOF-based microspheres for pulmonary delivery. The team reported a simple and convenient method for developing pulmonary-targeted composites based on MIL-100. For deep lung deposition, these MIL-100 systems were then encapsulated into microspheres with a spray drying technique using the negatively charged polysaccharide dextran, the oligosaccharide α-cyclodextrin, and the polyol mannitol. The *in vivo* studies done on rats showed that upon intratracheal administration, the system showed uniform release of the nano MIL-100 carrier inside the lungs, reaching the bronchioles and alveoli, proving that the MOF system can act as a potential means for pulmonary delivery [115]. Xiaoxiao Hu [116] presented a MOF platform for pulmonary delivery in a study they conducted. As a DPI (Dry Powder Inhaler) carrier for the drug budesonide (BUD), neutralized nanoporous cyclodextrin-MOF crystals with cubic morphology and uniform inhaling size were created and adjusted. To increase the flowability and particle aerodynamic behaviour of the CD-MOF powder, leucine (LEU)-poloxamer and cholesterol (CHO) were added, and the particle size distribution, Carr's index, and *in vitro* pulmonary deposition were measured as a result. CHO-CD-MOF outperformed CD-MOF or LEU-CD-MOF-BUD in terms of mass median aerodynamic diameter (4.35±0.04 μm) and inhalable performance (30.60±0.76 % fine particle percentage), both of which persisted after budesonide loading (4.47±0.30 μm, 24.95±4.33 %).Then, powder X-ray diffraction (PXRD), cell viability analysis, *in vivo* fluorescence imaging, and pharmacokinetic investigations in rats were used to assess the crystallinity, cytotoxicity, and *in vivo* deposition of drug-loaded materials (CHO-CD-MOF-BUD). After being loaded into CHO-CD-MOF, budesonide's distinctive PXRD crystallinity peaks vanished, possibly indicating that budesonide has been molecularly incorporated into the pores of CD-MOF. Due to CD-MOF's excellent biocompatibility, the A549 cell's viability for CHO-CD-MOF-BUD was greater than 90 %. The fluorescent signal at the lung tissue was significantly improved after cholesterol modification compared to CD-MOF when Rhodamine B was carried by the DPI particles, and the bioavailability of CHO-CD-MOF-BUD in rats was comparable to that of Pulmicort Turbuhaler, a commercial product. Therefore, the CD-MOF powders modified by cholesterol can be employed as a promising inhalable carrier for the pulmonary administration of medicines in tiny doses. In another study, CCM was loaded onto CD-MOFs in 2019 by Yixian Zhou [117] for pulmonary administration. CCM-loaded CD-MOFs (CCM-CDMOFs) had exceptional aerodynamic performance in comparison to micronized CCM generated by jet milling, which was attributed to the special porous structure and lower density of CD-MOFs. The dissolution test revealed that CCM-CD-MOFs released drugs at a rate that was significantly higher than that of micronized CCM. According to the all-atom molecular dynamic simulation, CCM molecules were found to be loaded into CD-MOFs' hydrophobic cavities or introduced into their vast hydrophilic cavities to form nanoclusters. The improved dissolution rate may be facilitated by the increased wettability of CCM-CD-MOFs and the distinctive spatial distribution feature of CCM in the porous interior of CD-MOFs. The DPPH radical scavenging assay demonstrated CCM-CD-MOFs' strong antioxidant capacity. Consequently, it was anticipated that CD-MOFs would be promising vehicles for the pulmonary administration of weakly water-soluble drugs. A series of homogenous nanopore-containing CD-MOF particles were expertly crafted without the use of porogens in a study by Yixian Zhou [118]. Due to its uniform nanoporous structure, CD-MOF demonstrated superior aerosolization ability compared to commercial inhalation carriers. A number of experiments, including cytotoxicity assays, hemolysis ratio tests, lung function assessments, *in-vivo* lung injury indicators measurements, and histological analyses, further supported CD-MOF's excellent biocompatibility in pulmonary delivery. The significant rate of CD-MOF deposition in the lungs was revealed by ex vivo fluorescence imaging data. In light of all the findings, CD-MOF was shown to be a potential carrier for pulmonary medication delivery. The nanoporous particles for efficient pulmonary delivery may be clarified by this investigation. In a similar line, Bader M. Jarai and colleagues [119] examined the application of the UiO-66 NPs for the targeted pulmonary drug delivery system. They evaluated the action of missing linker defects in pulmonary delivery. They determined that the missing linker defects resulted in a contrast in the aerodynamics of the NPs. On the other hand, it has a minimal effect on drug loading, drug release, biocompatibility and biodistribution. Also, the UiO-66-based system showed better pH-dependent stability and didn’t undergo degradation in extracellular conditions or breakdown in the intracellular environment. Further, the *in vitro* study suggested that it showed minimum toxicity and better biocompatibility, while the *in vivo* study showed good tolerance in murine evaluations after orotracheally administered NPs. The UiO-66 NPs remained in the lungs for seven days and showed localized action before clearance. Overall results showed that UiO-66 could act as a potential platform for pulmonary delivery owning to the localization, better release and biocompatibility. The volatile oil D-limonene (D-Lim), derived from citrus fruits, has anti-inflammatory and anti-cancer properties, but its physical instability makes deep delivery difficult. D-Lim was loaded into a biodegradable CD-MOF with an optimal loading efficiency of 13.79±0.01 % (the molar ratio of D-Lim and -CD-MOF was 1.6:1), which had cubic shape and controllable particle size (1-5 m) in order to prevent the volatilization of D-Lim and achieve effective pulmonary delivery. According to the experimental findings, -CD-MOF could increase D-Lim's stability. The interaction between D-Lim and -CD-MOF was discovered using several characterizations and molecular docking. The utilisation of D-Lim's inhalable dose form, dry powder inhaler (DPI), was greatly aided by the solidification of D-Lim by -CD-MOF. D-Lim@-CD-MOF was successfully aerosolized for inhalation with a fine particle fraction (FPF) of 33.12 ± 1.50 % at a flow rate of 65 L min^-1^. Additionally, an *in vivo* investigation revealed that D-Lim solidified by -CD-MOF for inhalation had a 2.23-fold higher bioavailability than D-Lim for oral delivery. As a result, it is thought that -CD-MOF could be a great drug delivery system for the lungs to achieve the solidification and therapeutic benefits of volatile oils [120]. While exploring the different means of administering MOF and its use in the arena of cosmetics, Alfonso García Márqueza *et al.* [121] proposed a convenient and eco-friendly method for the preparation of cutaneous patches comprising of drug nanocarrier MIL-100(Fe) and biopolymers for cosmetic applications. The ex-vivo and *in-vitro* models showed better release capabilities and encapsulation due to better physicochemical properties like swelling properties, structure, bio-adhesion, and hydration. These were evaluated using active pharmaceutical ingredients (APIs) like caffeine and IBU. Particularly, very large caffeine loadings were held within these cutaneous devices, with gradual releases occurring as a result of the hydrophilic patches expanding in simulated cutaneous physiological circumstances. These patches offered a gradual and appropriate penetration of their aesthetic payload through the skin, curiously reaching the targeted adipose tissue despite lacking any cutaneous bio-adhesive character. This makes these composite MOF-based patches for aesthetic applications, which include cosmetics, promising possibilities. In another similar work by Sara Rojas [122] on understanding the cutaneous administration and release of MOFs, a mixture of experimental and computational examinations was carried out to investigate the main physicochemical and structural parameters involved in the drug abortion kinetics of IBU and aspirin. For obtaining a variety of drug-matrix complexes with varying structural and physicochemical characteristics along with these two drugs, three model MOF systems were selected viz MIL-100(Fe), UiO-66(Zr), and MIL-127(Fe). This study proved that structural parameters and MOF/drug hydrophobic/hydrophilic balance are the key mechanisms governing drug loading and release. These devices were shown to meet the criteria for usage as topical drug-delivery systems, such as releasing the payload between 1 and 7 days by delivering the drug under simulated cutaneous conditions (aqueous medium at 37 °C). These findings demonstrate the significance of selecting MOFs carefully by demonstrating how the geometrical and chemical properties of both the MOF and the drug affect the adsorption and release of the drug. In another study, Sara Rojas and Patricia Horcajada [123] synthesized eight different MOF platforms for the topical delivery of salicylic acid to tackle the stability issue. Among the different structures, the microporous Zr amino terephthalate UiO-66-NH2 demonstrated the most promising results for the loading of salicylic acid. When the delivery procedure was examined in a simulated cutaneous condition comprising aqueous media at 37 °C, the maximum plasma concentration was reached in 6 h with a 64 % release. These findings show that UiO-66-NH_2_ is a viable option for the development of novel skin-treatment devices since it is suitable for the topical controlled release of SA. In another study, Mubarak G. Bello [124] used an easy green chemistry method to create a 2D nanosheet (NS) of CD-MOFs. The water system was used to create the NS-MOF carrier in a straightforward one-pot synthesis combining CD and potassium carbonate. By changing the reaction temperature and adding a crystal growth inhibitor (the proper amount of acetone), particle size optimisation is accomplished. The crosslinked CD-MOF (CL-CD-MOF) polymerization procedure increases the stability of NS-MOF in aqueous medium without obstructing the cavity for drug loading. In order to assess the impact of particle geometry and size on the pharmacokinetics of nanoporous materials during drug delivery, the same amount of dexamethasone (DXM) was loaded into both the sheetlike DXM@CL-NS-MOF and the 3D-cubic-shaped DXM@CL-CD-MOF. *In vivo* research is done to determine how well these carriers transport DXM through the tear and aqueous humours. The outcomes show that the 2D-nanosheet particles greatly outperform the commercial Maxidex (0.1 % dexamethasone) and its 3D-cube counterparts of similar chemical composition in terms of precorneal residence duration and intraocular bioavailability. It implies that a carrier's geometry affects biodistribution significantly, and the CL-NS-MOF carrier is an excellent choice for ocular drug administration. Similar research was done by Gandara-Loe and colleagues [125]. Zr-based UiO-67 as a MOF and polyurethane (PU) as a polymeric matrix was used to successfully construct novel MOF-based polymer nanocomposite films. The enhanced stability of the UiO-67 implanted nanocrystals is confirmed by synchrotron X-ray powder diffraction (SXRPD) analysis, and their homogeneous distribution (average crystal size of 100-200 nm) inside the 50 μm thick film is confirmed by SEM images. For N_2_ at cryogenic temperatures, access to the implanted MOFs' internal porous structure was prevented. At least 45 % of the MOF crystals are fully accessible for the gas phase adsorption of non-polar molecules, according to ethylene adsorption studies at 25 °C. Although this partial blocking hinders the embedded MOFs' ability to adsorb ocular medications (such as brimonidine tartrate) as compared to the pure MOF, the integration of UiO-67 nanocrystals increased the PU matrix's adsorption capacity by about 60 times. Brimonidine is released from UiO-67@PU nanocomposite over an extended time (up to 14 days were measured). The presence of the drug in the nanocomposite film, the stability of the MOF framework and the drug upon loading, and the existence of brimonidine in an amorphous phase after adsorbed were all validated by the combined use of SXRPD, TGA, and FTIR analysis. These findings pave the way for the use of these polymeric nanocomposite films for drug administration in optical treatments, whether as an element of contact lenses, a component of lacrimal stoppers (such as punctal plugs), or as sub-tenon inserts.

### Drug release mechanism

#### Conventional release

It's important to note that the drug release mechanism from MOFs can be influenced by various factors, including the specific MOF structure, pore size, drug loading method, and the physicochemical properties of the drug molecules. Therefore, it's crucial to consider these factors and conduct experimental studies to understand and optimize the drug release behaviour of MOFs for specific applications [126]. Drugs are essentially released from the MOF by breaking the bonds between metal ions and linkers, which causes the coordination structure to collapse and further releases the drug molecules at the precise location. The conventional drug release is independent or lacks target-specific drug delivery because it is not predicated on any particular stimuli [127].

#### Drug release kinetic models

The drug release from any drug delivery carrier is crucial, as it determines the efficacy of the dosage form. To study the pattern of drug release, researchers used different types of drug release kinetic models such as first order, zero order, Hixon-Crowell, Higuchi matrix, Korsmeyer-Peppas, Peppas-Sahlin, Baker-Lonsdale, *etc*. [128]. As porous MOFs are used for drug delivery purposes, their developed formulations are also evaluated for their drug release pattern. For example, Horcajada *et al.* [129] developed the nanoporous MIL-53 (Fe, Cr) for the delivery of IBU. The delivery of IBU was demonstrated using simulated body fluid (SBF) at 37 °C under continuous stirring, which was further analysed using HPLC. Unexpectedly, a very slow delivery that takes three weeks to finish is seen. There are clearly two processes involved. The data were treated as a first approximation and as having zero-order kinetics, as reported *(R^2^* = 0.99), regardless of minor changes in slope. In another study, Maryam Esfahanian *et al.* [130] reported a Fe_3_O_4_@PAA@ZIF-8 nanocarrier for the effective delivery of ciprofloxacin (CIP) for antibacterial activity against Staphylococcus aureus (S. aureus) and *Escherichia coli* (*E. coli*). The CIP release from nanocomposite was carried out in buffers pH 5 and 7.4. The developed nanocomposite was evaluated for different kinetic models such as zero-order, first-order, the Higuchi equation, and the Korsmeyer-Peppas equation. As far as results are concerned, the Higuchi and Peppas models are the best-fitted kinetic models. In a similar vein of work, Mohaddeseh Nasrabadi and his team [131] reported a CIP-UiO-66 nanoframework for the effective delivery of CIP for anti-bacterial activity. The drug release studies were performed in phosphate buffer pH 7.4 and acetate buffer pH 5.0. Furthermore, the data obtained from the CIP release was further evaluated for zero order, first order, Higuchi, Hixson, and Kor's Peppas kinetic models. On the basis of the findings, the release kinetic process of CIP from UiO-66 follows the Higuchi model and Kor's Peppas at pH 7.4 and 5.0. Khaled AbouAitah and co-workers [111] fabricated Zr-MOF (UiO-66-COOH) and titanium-based MOF (TiMOF) (MIL-125-NH_2_). They developed nanocarriers used for plant-based medicine delivery: Ru-flavonoid and Pip-alkaloid, which exhibit anti-oxidant and anti-inflammatory effects. Ru and Pip exhibited a zero-order release pattern for the initial 12 hours and further continued to follow the stable, sustained release. At pH 7.4, zero-order and Korsmeyer-Peppas kinetic models are the best fits for the release patterns. A. Bazzazzadeh and his research team [132] developed a magnetically responsive MIL-53 nanocarrier into core-shell nanofibers composed of polyacrylic acid-grafted chitosan and polyurethane (PA-g-CS/PU). For the controlled release delivery of temozolomide (TMZ) and PTX against U-87 MG glioblastoma cells during the chemotherapy/hyperthermia combined approach. Researchers investigated the effect of the hyperthermia phenomenon on the drug release pattern of loaded drugs. The non-Fickian diffusion of the Korsmeyer-Peppas equation provided the best explanation for the release data. In another work, Xin Leng and co-workers [133] developed a nanocarrier for efficient delivery of oridon (Ori)-MIL-53 (Fe) against liver cancer therapy. The research work comprised the synthesis and fabrication of MIL-53(Fe) nanocarriers along with different characterizations to evaluate their efficiency in carrying Ori selectively to liver cancer cells. The nanosystem was further evaluated for the drug release performance test; the evaluation was carried out in phosphate-buffered saline at pH 7.2 and 5.5, and findings revealed that the Ori release lasted for seven days. Ori-MIL-53 (Fe) followed the sustained release kinetic model for drug release; thus, it could be a promising candidate for sustained release drug delivery for anticancer applications.

## Current challenges and future prospects

In the present review, the MOFs' potential as intelligent theranostic nanosystems has been highlighted. These systems have excellent features such as high drug loading, controlled cargo release, surface modification, and stimulus responsiveness. The article also highlighted the synthesis and characterization techniques, cargo loading methodologies, formulation development aspects, non-stimuli and stimuli-responsive MOFs, drug release mechanism, and routes for delivering the MOFs [134]. The MOFs have a tunable surface architecture, which makes them promising carriers for targeted delivery, gene therapy, antibiotic delivery, and especially cancer targeting. Even though the study of MOF-based DDs has advanced quickly, numerous problems still need to be resolved before they can be used in clinical settings [135]. The biostability of MOFs is an important concern, as they need to be stable in the body so that they do not degrade before they release the drug. Toxicity-wise, MOFs need to be non-toxic to cells and tissues. From a manufacturing viewpoint, MOFs should be inexpensive to be used for wider clinical applications. These aforementioned hurdles need to be addressed before the successful stabilisation of MOFs in clinical implementation [136].

## Concluding remarks

In recent years, MOFs have secured a prime position as drug delivery carriers. The galoric MOF developments can be attributed to their high porosity and surface area, structural flexibility, and diverse functionality, leading to high drug loading and efficient drug release. Considering the potential of MOFs, the current review provides insights into structural composition, conventional and novel synthesis methods, characterization techniques, drug loading approaches, and formulation development strategies. Also, the distinction between non-responsive and stimuli-responsive MOFs and their role in drug delivery has been exhaustively discussed. Considering the distinct merits of MOFs, several routes, viz. oral, parenteral, *etc.*, have been explored for the administration of MOFs. Moreover, the favourable routes for MOF administration and drug release kinetics have been discussed in detail, providing in-depth peculiarities to serve as a potential candidate in the upcoming years of drug delivery. Additionally, MOF-based formulation development and optimisation considering DoE have been discussed thoroughly, which provides a platform for future considerations for formulation development. In brief, the growing interest in the scientific fraternity for MOF application in the biomedical field has provided many economical and green techniques for constructing MOFs. Also, by employing analytical techniques, the structural and morphological peculiarities of MOFs can be revealed and modified accordingly. The distinct analytical techniques give an idea of the versatility of MOFs in a series of applications, which primarily include drug delivery aspects. In terms of drug loading, several strategies have been offered to ensure uniform drug distribution. These strategies furnish the suitability of methods according to the type of material used for the entrapment. Furthermore, the stimuli-responsive potential of MOFs can efficiently provide site-specific drug delivery. Stimuli-responsive MOFs have several benefits, such as precise targeting of actives, controlled drug release, high drug loading ability, and the utilisation of altered physiological changes to target relatable diseases. Along with the insights of stimuli-responsive MOFs, the multiples of diseases with their background for development, targetability, and future propensity in clinical applications were elaborated thoroughly. The routes of administration offer a comprehensive aspect of several routes in alliance with different experimental research, which would be beneficial while ensuring the safe targeting of actives via MOFs. As a result, the multifunctional potential of MOFs can potentially offer a great avenue for drug delivery applications. Even though some important aspects related to their biosafety, off-target accumulation and manufacturing setting should be critical considerations.

## References

[1] Adepu S. Ramakrishna S.. Controlled Drug Delivery Systems: Current Status and Future Directions. Molecules 26 (2021) 5905. https://doi.org/10.3390/molecules26195905 10.3390/molecules26195905 34641447 PMC8512302

[2] Wang Y. Yan J. Wen N. Xiong H. Cai S. He Q. Hu Y. Peng D. Liu Z. Liu Y.. Metal-Organic Frameworks for Stimuli-Responsive Drug Delivery. Biomaterials 230 (2020) 119619. https://doi.org/10.1016/j.biomaterials.2019.119619 10.1016/j.biomaterials.2019.119619 31757529

[3] Tibbetts I. Kostakis G.E.. Recent Bio-Advances in Metal-Organic Frameworks. Molecules 25 (2020) 1291. https://doi.org/10.3390/molecules25061291 10.3390/molecules25061291 32178399 PMC7144006

[4] Zhang S. Pei X. Gao H. Chen S. Wang J.. Metal-Organic Framework-Based Nanomaterials for Biomedical Applications. Chinese Chemical Letters 31 (2020) 1060-1070. https://doi.org/10.1016/j.cclet.2019.11.036 10.1016/j.cclet.2019.11.036

[5] Mohammadi S. Z. Mousazadeh F. Mohammadhasani-Pour M.. Electrochemical Detection of Folic Acid Using a Modified Screen Printed Electrode. Journal of Electrochemical Science and Engineering 12 (2022) 1111-1120. https://doi.org/10.5599/jese.1360 10.5599/jese.1360

[6] Pantwalawalkar J. Mhettar P. Nangare S. Mali R. Ghule A. Patil P. Mohite S. More H. Jadhav N.. Stimuli-Responsive Design of Metal-Organic Frameworks for Cancer Theranostics: Current Challenges and Future Perspective. ACS Biomaterial Science & Engineering 9(8) (2023) 4497-4626. https://doi.org/10.1021/acsbiomaterials.3c00507 10.1021/acsbiomaterials.3c00507 37526605

[7] Anumah A. Louis H. Hamzat A.T. Amusan O.O. Pigweh A.I. Akakuru O.U. Adeleye A.T. Magu T.O., Chemical Methodologies Metal-Organic Frameworks (MOFs): Recent Advances in Synthetic Methodologies and Some Applications. Chemical Methodologies 3 (2019) 283-305. http://dx.doi.org/10.22034/CHEMM.2018.139807.1067 10.22034/CHEMM.2018.139807.1067

[8] Maranescu B. Visa A.. Applications of Metal-Organic Frameworks as Drug Delivery Systems. International Journal of Molecular Sciences 23(8) (2022) 1-19. https://doi.org/10.3390/ijms23084458 10.3390/ijms23084458 PMC9026733 35457275

[9] Al Sharabati M. Sabouni R. Husseini G.A.. Biomedical Applications of Metal−Organic Frameworks for Disease Diagnosis and Drug Delivery: A Review. Nanomaterials 12(2) (2022) 277. https://doi.org/10.3390/nano12020277 10.3390/nano12020277 35055294 PMC8780624

[10] Osterrieth J.W.M. Fairen-Jimenez D.. Metal-Organic Framework Composites for Theragnostics and Drug Delivery Applications. Biotechnology Journal 16(2) (2021) 2000005. https://doi.org/10.1002/biot.202000005 10.1002/biot.202000005 32330358

[11] Rahaman S.J. Samanta A. Mir M.H. Dutta B.. Metal-Organic Frameworks (MOFs): A Promising Candidate for Stimuli-Responsive Drug Delivery. ES Materials and Manufacturing 19 (2023) 792. https://doi.org/10.30919/esmm5f792 10.30919/esmm5f792

[12] Raptopoulou C.P.. Metal-Organic Frameworks: Synthetic Methods and Potential Applications. Materials 14(2) (2021) 310. https://doi.org/10.3390/ma14020310 10.3390/ma14020310 33435267 PMC7826725

[13] Yusuf V.F. Malek N.I. Kailasa S.K.. Review on Metal-Organic Framework Classification, Synthetic Approaches, and Influencing Factors: Applications in Energy, Drug Delivery, and Wastewater Treatment. ACS Omega 7(49) (2022) 44507-44531. https://doi.org/10.1021/acsomega.2c05310 10.1021/acsomega.2c05310 36530292 PMC9753116

[14] Hidalgo T. Serre C. Horcajada P.. Applications. Nanostructured metal-organic frameworks and their bio-related applications. Coordination Chemistry Review 307 (2015) 342-360. https://doi.org/10.1016/j.ccr.2015.08.008 10.1016/j.ccr.2015.08.008

[15] Sohrabi H. Javanbakht S. Oroojalian F. Rouhani F. Shaabani A. Majidi M.R. Hashemzaei M. Hanifehpour Y. Mokhtarzadeh A. Morsali A.. Nanoscale Metal-Organic Frameworks: Recent Developments in Synthesis, Modifications and Bioimaging Applications. Chemosphere 281 (2021) 130717 https://doi.org/10.1016/j.chemosphere.2021.130717 10.1016/j.chemosphere.2021.130717 34020194

[16] Remya V.R. Kurian M.. Synthesis and Catalytic Applications of Metal-Organic Frameworks: A Review on Recent Literature. International Nano Letters 9 (2019) 17-29. https://doi.org/10.1007/s40089-018-0255-1 10.1007/s40089-018-0255-1

[17] Liu L. Wei H. Zhang L. Li J. Dong J.. Ionothermal Synthesis of the Metal-Organic Framework Compound Cu3(BTC)2. Studies in Surface Science and Catalysis 14 (2008) 459-462. https://doi.org/10.1016/S0167-2991(08)80240-6 10.1016/S0167-2991(08)80240-6

[18] Sharanyakanth P.S. Radhakrishnan M.. Synthesis of Metal-Organic Frameworks (MOFs) and Its Application in Food Packaging: A Critical Review. Trends Food Science Technology 104 (2020) 102-116. https://doi.org/10.1016/j.tifs.2020.08.004 10.1016/j.tifs.2020.08.004

[19] Bedia J. Muelas-Ramos V. Peñas-Garzón M. Gómez-Avilés A. Rodríguez J.J. Belver C.. A Review on the Synthesis and Characterization of Metal Organic Frameworks for Photocatalytic Water Purification. Catalysts 9(1) (2019) 52. https://doi.org/10.3390/catal9010052 10.3390/catal9010052

[20] Lee Y.R. Kim J. Ahn W.S.. Synthesis of Metal-Organic Frameworks: A Mini Review. Korean Journal of Chemical Engineering. 30(9) (2013) 1667-1680. https://doi.org/10.1007/s11814-013-0140-6 10.1007/s11814-013-0140-6

[21] Echaide-Górriz C. Clément C. Cacho-Bailo F. Téllez C. Coronas J.. New Strategies Based on Microfluidics for the Synthesis of Metal Organic Frameworks. Journal of Material Chemistry A 6(14) (2018) 5485-5506. https://doi.org/10.1039/C8TA01232F 10.1039/C8TA01232F

[22] Stock N. Biswas S.. Synthesis of Metal-Organic Frameworks (MOFs): Routes to Various MOF Topologies, Morphologies, and Composites. Chemical Reviews 112(2) (2012) 933-969. https://doi.org/10.1021/cr200304e 10.1021/cr200304e 22098087

[23] Varsha V.M. Nageswaran G.. Review—Direct Electrochemical Synthesis of Metal Organic Frameworks. Journal of Electrochemical Society 167 (2020) 155527. https://doi.org/10.1149/1945-7111/abc6c6 10.1149/1945-7111/abc6c6

[24] Bag P.P. Singh G.P. Singha S. Roymahapatra G.. Synthesis of Metal-Organic Frameworks (MOFs) and Their Biological, Catalytic and Energetic Applications. Engineered Science 13 (2021) 1-10. https://doi.org/10.30919/es8d1166 10.30919/es8d1166

[25] Sun Y. Zheng L. Yang Y. Qian X. Fu T. Li X. Yang Z. Yan H. Cui C. Tan W.. Metal-Organic Framework Nanocarriers for Drug Delivery in Biomedical Applications. Nano-Micro Letters 12(1) (2020) 1-29. https://doi.org/10.1007/s40820-020-00423-3 10.1007/s40820-020-00423-3 PMC7770922 34138099

[26] Vodyashkin A.A. ergorodceva A.V. Kezimana P. Stanishevskiy Y.M.. Metal-Organic Framework (MOF)—A Universal Material for Biomedicine. International Journal of Molecular Sciences 24(9) (2023) 7819. https://doi.org/10.3390/ijms24097819 10.3390/ijms24097819 37175523 PMC10178275

[27] Kalaj M. Cohen S.M.. Postsynthetic Modification: An Enabling Technology for the Advancement of Metal-Organic Frameworks. ACS Central Science 7 (2020) 1046-1057. https://doi.org/10.1021/acscentsci.0c00690 10.1021/acscentsci.0c00690 PMC7379093 32724840

[28] Rivera-Torrente M. Mandemaker L.D.B. Filez M. Delen G. Seoane B. Meirer F. Weckhuysen B.M.. Spectroscopy, Microscopy, Diffraction and Scattering of Archetypal MOFs: Formation, Metal Sites in Catalysis and Thin Films. Chemical Society Reviews 49 (2020) 6694-6732. https://doi.org/10.1039/d0cs00635a 10.1039/d0cs00635a 32820300

[29] Butova V.V Soldatov M.A. Guda A.A. Lomachenko K.A. Lamberti C.. Metal-Organic Frameworks: Structure, Properties, Methods of Synthesis and Characterization. Russian Chemical Reviews 3 (2016) 280-307. https://doi.org/10.1070/rcr4554 10.1070/rcr4554

[30] Lázaro I.A.. A Comprehensive Thermogravimetric Analysis Multifaceted Method for the Exact Determination of the Composition of Multifunctional Metal-Organic Framework Materials. European Journal of Inorganic Chemistry 45 (2020) 4284-4294. https://doi.org/10.1002/ejic.202000656 10.1002/ejic.202000656

[31] Howarth A.J. Peters A.W. Vermeulen N.A. Wang T.C. Hupp J.T. Farha O.K.. Best Practices for the Synthesis, Activation, and Characterization of Metal−organic Frameworks. Chemistry of Materials 29(1) (2017) 26-39. https://doi.org/10.1021/acs.chemmater.6b02626 10.1021/acs.chemmater.6b02626

[32] Hirschle P. Preiß T. Auras F. Pick A. Völkner J. Valdepérez D. Witte G. Parak W.J. Rädler J.O. Wuttke S.. . Exploration of MOF Nanoparticle Sizes Using Various Physical Characterization Methods-Is What You Measure What You Get? CrystEngComm 23 (2016) 4359-4368. https://doi.org/10.1039/c6ce00198j 10.1039/c6ce00198j

[33] Marsh C. Shearer G.C. Knight B.T. Paul-Taylor J. Burrows A.D.. Supramolecular Aspects of Biomolecule Interactions in Metal-Organic Frameworks. Coordination Chemistry Reviews 439 (2021) 213928. https://doi.org/10.1016/j.ccr.2021.213928 10.1016/j.ccr.2021.213928

[34] Lucier B.E.G. Chen S. Huang Y.. Characterization of Metal-Organic Frameworks: Unlocking the Potential of Solid-State NMR. Accounts of Chemical Research 2 (2018) 319-330. https://doi.org/10.1021/acs.accounts.7b00357 10.1021/acs.accounts.7b00357 29251909

[35] Wei Y.S. Zhang M. Zou R. Xu Q.. Metal-Organic Framework-Based Catalysts with Single Metal Sites. Chemical Reviews 121(21) (2020) 12089-12174. https://doi.org/10.1021/acs.chemrev.9b00757 10.1021/acs.chemrev.9b00757 32356657

[36] Hadjiivanov K.I. Panayotov D.A. Mihaylov M.Y. Ivanova E.Z. Chakarova K.K. Andonova S.M. Drenchev N.L.. Power of Infrared and Raman Spectroscopies to Characterize Metal-Organic Frameworks and Investigate Their Interaction with Guest Molecules. Chemical Reviews 121(3) (2021) 1286-1424. https://doi.org/10.1021/acs.chemrev.0c00487 10.1021/acs.chemrev.0c00487 33315388

[37] He S. Wu L. Li X. Sun H. Xiong T. Liu J. Huang C. Xu H. Sun H. Chen W. Gref R. Zhang J.. Metal-Organic Frameworks for Advanced Drug Delivery. Acta Pharmaceutica Sinica B 11(8) (2021) 2362-2395. https://doi.org/10.1016/j.apsb.2021.03.019 10.1016/j.apsb.2021.03.019 34522591 PMC8424373

[38] Wen T. Quan G. Niu B. Zhou Y. Zhao Y. Lu C. Pan X. Wu C.. Versatile Nanoscale Metal-Organic Frameworks (NMOFs): An Emerging 3D Nanoplatform for Drug Delivery and Therapeutic Applications. Small 17(8) (2021) 2005064. https://doi.org/10.1002/smll.202005064 10.1002/smll.202005064 33511778

[39] Lawson H.D. Walton S.P. Chan C.. Metal-Organic Frameworks for Drug Delivery: A Design Perspective. ACS Applied Materials& Interfaces 6 (2021) 7004-7020. https://doi.org/10.1021/acsami.1c01089 10.1021/acsami.1c01089 PMC11790311 33554591

[40] Zheng H. Zhang Y. Liu L. Wan W. Guo P. Nyström A.M. Zou X.. One-Pot Synthesis of Metal-Organic Frameworks with Encapsulated Target Molecules and Their Applications for Controlled Drug Delivery. Journal of the American Chemical Society 3 (2016) 962-968. https://doi.org/10.1021/jacs.5b11720 10.1021/jacs.5b11720 26710234

[41] Kritskiy I. Volkova T. Surov A. Terekhova I.. γ-Cyclodextrin-Metal Organic Frameworks as Efficient Microcontainers for Encapsulation of Leflunomide and Acceleration of Its Transformation into Teriflunomide. Carbohydrate Polymers 216 (2019) 224-230. https://doi.org/10.1016/j.carbpol.2019.04.037 10.1016/j.carbpol.2019.04.037 31047061

[42] Vassaki M. Papathanasiou K.E. Hadjicharalambous C. Chandrinou D. Turhanen P. Choquesillo-Lazarte D. Demadis K.D.. Self-Sacrificial MOFs for Ultra-Long Controlled Release of Bisphosphonate Anti-Osteoporotic Drugs. Chemical Communications 238 (2020) 5166-5169. https://doi.org/10.1039/d0cc00439a 10.1039/d0cc00439a 32255461

[43] Li S. Huo F.. Metal-Organic Framework Composites: From Fundamentals to Applications. Nanoscale 7(17) (2015) 7482-7501. https://doi.org/10.1039/c5nr00518c 10.1039/c5nr00518c 25871946

[44] Devautour-Vinot S. Martineau C. Diaby S. Ben-Yahia M. Miller S. Serre C. Horcajada P. Cunha D. Taulelle F. Maurin G.. Caffeine Confinement into a Series of Functionalized Porous Zirconium MOFs: A Joint Experimental/Modeling Exploration. Journal of Physical Chemistry C 22 (2013) 11694-11704. https://doi.org/10.1021/jp402916y 10.1021/jp402916y

[45] Nadizadeh Z. Naimi-Jamal M.R. Panahi L.. Mechanochemical Solvent-Free in Situ Synthesis of Drug-Loaded {Cu2(1,4-Bdc)2(Dabco)}n MOFs for Controlled Drug Delivery. Journal of Solid State Chemistry 259 (2018) 35-42. https://doi.org/10.1016/j.jssc.2017.12.027 10.1016/j.jssc.2017.12.027

[46] Noorian S.A. Hemmatinejad N. Navarro J.A.R.. Bioactive Molecule Encapsulation on Metal-Organic Framework via Simple Mechanochemical Method for Controlled Topical Drug Delivery Systems. Microporous & Mesoporous Materials 302 (2020) 110199. https://doi.org/10.1016/j.micromeso.2020.110199 10.1016/j.micromeso.2020.110199

[47] Morris W. Briley W.E. Auyeung E. Cabezas M.D. Mirkin C.A.. Nucleic Acid-Metal Organic Framework (MOF) Nanoparticle Conjugates. Journal of American Chemical Society 20 (2014) 7261-7264. https://doi.org/10.1021/ja503215w 10.1021/ja503215w 24818877

[48] Giménez-Marqués M. Hidalgo T. Serre C. Horcajada P.. Nanostructured Metal-Organic Frameworks and Their Bio-Related Applications. Coordination Chemistry Reviews 307 (2016) 342-360. https://doi.org/10.1016/j.ccr.2015.08.008 10.1016/j.ccr.2015.08.008

[49] Anastasiadis S. H. Chrissopoulou K. Stratakis E. Kavatzikidou P. Kaklamani G. Ranella A.. How the Physicochemical Properties of Manufactured Nanomaterials Affect Their Performance in Dispersion and Their Applications in Biomedicine: A Review. Nanomaterials 12(3) (2022) 552. https://doi.org/10.3390/nano12030552 10.3390/nano12030552 35159897 PMC8840392

[50] McKinlay A.C. Morris R.E. Horcajada P. Férey G. Gref R. Couvreur P. Serre C.. BioMOFs: Metal-Organic Frameworks for Biological and Medical Applications. AngewandteChemie - International Edition 49(36) (2010) 6260-6266. https://doi.org/10.1002/anie.201000048 10.1002/anie.201000048 20652915

[51] Lamberti F. Mazzariol C. Spolaore F. Ceccato R. Salmaso L. Gross S.. Design of Experiment: A Rational and Still Unexplored Approach to Inorganic Materials’ Synthesis. Sustainable Chemistry 1 (2022) 114-130. https://doi.org/10.3390/suschem3010009 10.3390/suschem3010009

[52] Tavares Luiz M. Santos Rosa Viegas J. Palma Abriata J. Viegas F. Testa Moura de Carvalho Vicentini F. Lopes Badra Bentley M.V. Chorilli M. Maldonado Marchetti J. Tapia-Blácido D.R.. Design of Experiments (DoE) to Develop and to Optimize Nanoparticles as Drug Delivery Systems. European Journal of Pharmaceutics and Biopharmaceutics 165 (2021) 127-148. https://doi.org/10.1016/j.ejpb.2021.05.011 10.1016/j.ejpb.2021.05.011 33992754

[53] Li Y. Li X. Guan Q. Zhang C. Xu T. Dong Y. Bai X. Zhang W.. Strategy for Chemotherapeutic Delivery Using a Nanosized Porous Metal-Organic Framework with a Central Composite Design. International Journal of Nanomedicine 12 (2017) 1465-1474. https://doi.org/10.2147/IJN.S119115 10.2147/IJN.S119115 28260892 PMC5328663

[54] Kush P. Madan J. Kumar P.. Application Of Central Composite Design And Response Surface Methodology For Optimization Of Metal Organic Framework: Novel Carrier For Drug Delivery. Asian Journal of Pharmaceutical and Clinical Research 12(8) (2019) 121-127. https://doi.org/10.22159/ajpcr.2019.v12i8.34299 10.22159/ajpcr.2019.v12i8.34299

[55] He S. Wu L. Li X. Sun H. Xiong T. Liu J. Huang C. Xu H. Sun H. Chen W. Gref R. Zhang J.. Metal-Organic Frameworks for Advanced Drug Delivery. Acta Pharmaceutica Sinica B 11(8) (2021) 2362-2395. https://doi.org/10.1016/j.apsb.2021.03.019 10.1016/j.apsb.2021.03.019 34522591 PMC8424373

[56] Tamames-Tabar C. García-Márquez A. Blanco-Prieto M. J. Serre C. Horcajada P. Ruiz-Molina D. Novio F. Roscini C.. MOFs in Pharmaceutical Technology. Bio and Bioinspired Nanomaterials (2014) 83-112. http://dx.doi.org/10.1002/9783527675821.ch04 10.1002/9783527675821.ch04

[57] Karimzadeh S. Javanbakht S. Baradaran B. Shahbazi M.A. Hashemzaei M. Mokhtarzadeh A. Santos H.A.. Synthesis and Therapeutic Potential of Stimuli-Responsive Metal-Organic Frameworks. Chemical Engineering Journal 408 (2021) 127233. https://doi.org/10.1016/j.cej.2020.127233 10.1016/j.cej.2020.127233

[58] Carrillo-Carrión C.. Nanoscale Metal-Organic Frameworks as Key Players in the Context of Drug Delivery: Evolution toward Theranostic Platforms. Analytical and Bioanalytical Chemistry 412 (2020) 37-54. https://doi.org/10.1007/s00216-019-02217-y 10.1007/s00216-019-02217-y 31734711

[59] Liu Y. Zhao Y. Chen X.. Bioengineering of Metal-Organic Frameworks for Nanomedicine. Theranostics 9(11) (2019) 3122-3133. https://doi.org/10.7150/thno.31918 10.7150/thno.31918 31244945 PMC6567971

[60] Duan F. Feng X. Yang X. Sun W. Jin Y. Liu H. Ge K. Li Z. Zhang J.. A Simple and Powerful Codelivery System Based on PH-Responsive Metal-Organic Frameworks for Enhanced Cancer Immunotherapy. Biomaterials 122 (2017) 23-33. https://doi.org/10.1016/j.biomaterials.2017.01.017 10.1016/j.biomaterials.2017.01.017 28107662

[61] Zhang F.M. Dong H. Zhang X. Sun X.J. Liu M. Yang D.D. Liu X. Wei J.Z.. Postsynthetic Modification of ZIF-90 for Potential Targeted Codelivery of Two Anticancer Drugs. ACS Applied Material Interfaces 32 (2017) 27332-27337. https://doi.org/10.1021/acsami.7b08451 10.1021/acsami.7b08451 28745483

[62] Sun C.Y. Qin C. Wang X.L. Yang G.S. Shao K.Z. Lan Y.Q. Su Z.M. Huang P. Wang C.G. Wang E.B.. Zeolitic Imidazolate Framework-8 as Efficient PH-Sensitive Drug Delivery Vehicle. Dalton Transactions 41(23) (2012) 6906-6909. https://doi.org/10.1039/c2dt30357d 10.1039/c2dt30357d 22580798

[63] Guillen S.G. Parres-Gold J. Ruiz A. Lucsik E. Dao B. Hang T.K.L. Chang M. Garcia A.O. Wang Y. Tian F.. PH-Responsive Metal-Organic Framework Thin Film for Drug Delivery. Langmuir 38(51) (2022) 16014-16023. https://doi.org/10.1021/acs.langmuir.2c02497 10.1021/acs.langmuir.2c02497 36516863 PMC9798862

[64] Zhang Z.J. Hou Y.K. Chen M.W. Yu X.Z. Chen S.Y. Yue Y.R. Guo X.T. Chen J.X. Zhou Q.A.. PH-Responsive Metal-Organic Framework for the Co-Delivery of HIF-2α SiRNA and Curcumin for Enhanced Therapy of Osteoarthritis. Journal of Nanobiotechnology 21(1) (2021) 18. https://doi.org/10.1186/s12951-022-01758-2 10.1186/s12951-022-01758-2 PMC9847079 36650517

[65] Ai X. Mu J. Xing B.. Recent Advances of Light-Mediated Theranostics. Theranostics. 6(13) (2016) 2439-2457. https://doi.org/10.7150/thno.16088 10.7150/thno.16088 27877246 PMC5118606

[66] Rice A.M. Martin C.R. Galitskiy V.A. Berseneva A.A. Leith G.A. Shustova N.B.. Photophysics Modulation in Photoswitchable Metal-Organic Frameworks. Chemical Reviews 120(16) (2020) 8790-8813. https://doi.org/10.1021/acs.chemrev.9b00350 10.1021/acs.chemrev.9b00350 31638383

[67] Roth Stefaniak K. Epley C.C. Novak J.J. McAndrew M.L. Cornell H.D. Zhu J. McDaniel D.K. Davis J.L. Allen I.C. Morris A.J. Grove T.Z.. Photo-Triggered Release of 5-Fluorouracil from a MOF Drug Delivery Vehicle. Chemical Communications 55 (2018) 7617-7620. https://doi.org/10.1039/c8cc01601a 10.1039/c8cc01601a 29926872

[68] Cornell H.D. Morris A. Gandour R.D. Matson J. Tanko J.M.. A Green Light at the Intersection of Metal-Organic Frameworks and Drug Delivery. Chemical Communications 58 (2022) 5225-5228. https://doi.org/10.1039/D2CC00591C 10.1039/D2CC00591C 35380568

[69] Park J. Jiang Q. Feng D. Mao L. Zhou H.C.. Size-Controlled Synthesis of Porphyrinic Metal-Organic Framework and Functionalization for Targeted Photodynamic Therapy. Journal of American Chemical Society 10 (2016) 3518-3525. https://doi.org/10.1021/jacs.6b00007 10.1021/jacs.6b00007 26894555

[70] Liu Y. Liu Y. Bu W. Cheng C. Zuo C. Xiao Q. Sun Y. Ni D. Zhang C. Liu J. Shi J.. Hypoxia Induced by Upconversion-Based Photodynamic Therapy: Towards Highly Effective Synergistic Bioreductive Therapy in Tumors. Angewandte Chemie - International Edition 28 (2015) 8105-8109. https://doi.org/10.1002/anie.201500478 10.1002/anie.201500478 26012928

[71] Xu D. You Y. Zeng F. Wang Y. Liang C. Feng H. Ma X.. Disassembly of Hydrophobic Photosensitizer by Biodegradable Zeolitic Imidazolate Framework-8 for Photodynamic Cancer Therapy. ACS Applied Material& Interfaces 10(18) (2018), 15517-15523. https://doi.org/10.1021/acsami.8b03831 10.1021/acsami.8b03831 29677444

[72] Sharsheeva A. Iglin V.A. Nesterov P.V. Kuchur O.A. Garifullina E. Hey-Hawkins E. Ulasevich S.A. Skorb E.V. Vinogradov A.V. Morozov M.I.. Light-Controllable Systems Based on TiO2-ZIF-8 Composites for Targeted Drug Release: Communicating with Tumour Cells. Journal of Material Chemistry B7 43 (2019) 6810-6821. https://doi.org/10.1039/c9tb01377f 10.1039/c9tb01377f 31608920

[73] Fan Z. Liu H. Xue Y. Lin J. Fu Y. Xia Z. Pan D. Zhang J. Qiao K. Zhang Z. Liao Y.. Reversing Cold Tumors to Hot: An Immunoadjuvant-Functionalized Metal-Organic Framework for Multimodal Imaging-Guided Synergistic Photo-Immunotherapy. Bioactive Materials 6(2) (2021) 312-325. https://doi.org/10.1016/j.bioactmat.2020.08.005 10.1016/j.bioactmat.2020.08.005 32954050 PMC7475520

[74] Jiang K. Zhang L. Hu Q. Zhang Q. Lin W. Cui Y. Yang Y. Qian G.. Thermal Stimuli-Triggered Drug Release from a Biocompatible Porous Metal-Organic Framework. Chemistry - A European Journal 42 (2017) 10215-10221. https://doi.org/10.1002/chem.201701904 10.1002/chem.201701904 28682004

[75] Tian Y.Q. Chen Z.X. Weng L.H. Bin Guo H. Gao S. Zhao D.Y.. Two Polymorphs of Cobalt(II) Imidazolate Polymers Synthesized Solvothermally by Using One Organic Template N,N-Dimethylacetamide. Inorganic Chemistry 15 (2004) 4631-4635. https://doi.org/10.1021/ic049713z 10.1021/ic049713z 15257592

[76] Nagata S. Kokado K. Sada K.. Metal-Organic Framework Tethering PNIPAM for ON-OFF Controlled Release in Solution. Chemical Communications 51(41) (2015) 8614-8617. https://doi.org/10.1039/C5CC02339D 10.1039/C5CC02339D 25896867

[77] Mura S. Nicolas J. Couvreur P.. Stimuli-Responsive Nanocarriers for Drug Delivery. Nature Materials 12(11) (2013) 991-1003. https://doi.org/10.1038/nmat3776 10.1038/nmat3776 24150417

[78] Lin W. Hu Q. Yu J. Jiang K. Yang Y. Xiang S. Cui Y. Yang Y. Wang Z. Qian G.. Low Cytotoxic Metal-Organic Frameworks as Temperature-Responsive Drug Carriers. Chempluschem 81(8) (2016), 804-810. https://doi.org/10.1002/cplu.201600142 10.1002/cplu.201600142 31968821

[79] Silva J.Y.R. Proenza Y.G. da Luz L.L. de Sousa Araújo S. Filho M.A.G. Junior M.S.A. Soares T.A. Longo R.L.. A Thermo-Responsive Adsorbent-Heater-Thermometer Nanomaterial for Controlled Drug Release: (ZIF-8,EuxTby)@AuNP Core-Shell. Materials Science and Engineering C 102 (2019) 578-588. https://doi.org/10.1016/j.msec.2019.04.078 10.1016/j.msec.2019.04.078 31147030

[80] Wu W. Liu J. Gong P. Li Z. Ke C. Qian Y. Luo H. Xiao L. Zhou F. Liu W.. Construction of Core-Shell NanoMOFs@microgel for Aqueous Lubrication and Thermal-Responsive Drug Release. Small 18(28) (2022) 2202510. https://doi.org/10.1002/smll.202202510 10.1002/smll.202202510 35710878

[81] Li X.Y. Guan Q.X. Shang Y.Z. Wang Y.H. Lv S.W. Yang Z.X. Wang R. Feng Y.F. Li W.N. Li Y.J.. Metal-Organic Framework IRMOFs Coated with a Temperature-Sensitive Gel Delivering Norcantharidin to Treat Liver Cancer. World J Gastroenterology 27(26) (2021) 4208-4220. https://doi.org/10.3748/wjg.v27.i26.4208 10.3748/wjg.v27.i26.4208 PMC8311525 34326620

[82] Li J. Huo M. Wang J. Zhou J. Mohammad J.M. Zhang Y. Zhu Q. Waddad A.Y. Zhang Q.. Redox-Sensitive Micelles Self-Assembled from Amphiphilic Hyaluronic Acid-Deoxycholic Acid Conjugates for Targeted Intracellular Delivery of Paclitaxel. Biomaterials 7 (2012) 2310-2320. https://doi.org/10.1016/j.biomaterials.2011.11.022 10.1016/j.biomaterials.2011.11.022 22166223

[83] Zhao J. Yang Y. Han X. Liang C. Liu J. Song X. Ge Z. Liu Z.. Redox-Sensitive Nanoscale Coordination Polymers for Drug Delivery and Cancer Theranostics. ACS Applied Material & Interfaces 28 (2017) 23555-23563. https://doi.org/10.1021/acsami.7b07535 10.1021/acsami.7b07535 28636308

[84] Fukino T. Yamagishi H. Aida T.. Redox-Responsive Molecular Systems and Materials. Advanced Materials 29(25) (2017) 1603888. https://doi.org/10.1002/adma.201603888 10.1002/adma.201603888 27990693

[85] Ma A. Zhang R.. Facile Synthesis of Redox-Responsive Paclitaxel Drug Release Platform Using Metal-Organic Frameworks (ZIF-8) for Gastric Cancer Treatment. Material Research Express 7(9) (2020) 095402. https://doi.org/10.1088/2053-1591/abb2ce 10.1088/2053-1591/abb2ce

[86] Lei B. Wang M. Jiang Z. Qi W. Su R. He Z.. Constructing Redox-Responsive Metal-Organic Framework Nanocarriers for Anticancer Drug Delivery. ACS Applied Material & Interfaces 10(19) (2018) 16698-16706. https://doi.org/10.1021/acsami.7b19693 10.1021/acsami.7b19693 29692177

[87] He H. Du L. Guo H. An Y. Lu L. Chen Y. Wang Y. Zhong H. Shen J. Wu J. Shuai X.. Redox Responsive Metal Organic Framework Nanoparticles Induces Ferroptosis for Cancer Therapy. Small 16(33) (2020) 2001251. https://doi.org/10.1002/smll.202001251 10.1002/smll.202001251 32677157

[88] Huizenga D.E. Szostak J.W.. A DNA Aptamer That Binds Adenosine and ATP. Biochemistry 34(2) (1995) 656-665. https://doi.org/10.1021/bi00002a033 10.1021/bi00002a033 7819261

[89] Chen W.H. Yu X. Liao W.C. Sohn Y.S. Cecconello A. Kozell A. Nechushtai R. Willner I.. ATP-Responsive Aptamer-Based Metal-Organic Framework Nanoparticles (NMOFs) for the Controlled Release of Loads and Drugs. Advanced Functional Materials 27(37) (2017) 1702102. https://doi.org/10.1002/adfm.201702102 10.1002/adfm.201702102

[90] Chen W.H. Liao W.C. Sohn Y.S. Fadeev M. Cecconello A. Nechushtai R. Willner I.. Stimuli-Responsive Nucleic Acid-Based Polyacrylamide Hydrogel-Coated Metal-Organic Framework Nanoparticles for Controlled Drug Release. Advanced Functional Material 28(8) (2018) 1705137. https://doi.org/10.1002/adfm.201705137 10.1002/adfm.201705137

[91] Yang X. Tang Q. Jiang Y. Zhang M. Wang M. Mao L.. Nanoscale ATP-Responsive Zeolitic Imidazole Framework-90 as a General Platform for Cytosolic Protein Delivery and Genome Editing. Journal of American Chemical Society 141(9) (2019) 3782-3786. https://doi.org/10.1021/jacs.8b11996 10.1021/jacs.8b11996 30722666

[92] Lin W. Cui Y. Yang Y. Hu Q. Qian G.. A Biocompatible Metal-Organic Framework as a PH and Temperature Dual-Responsive Drug Carrier. Dalton Transactions 47 (2018) 15882-15887. https://doi.org/10.1039/c8dt03202e 10.1039/c8dt03202e 30362496

[93] Tan L.L. Song N. Zhang S.X.A. Li H. Wang B. Yang Y.W.. Ca2+, PH and Thermo Triple-Responsive Mechanized Zr-Based MOFs for on-Command Drug Release in Bone Diseases. Journal of Material Chemistry B 4(1) (2016) 135-140. https://doi.org/10.1039/c5tb01789k 10.1039/c5tb01789k 32262817

[94] Nagata S. Kokado K. Sada K.. Metal-Organic Framework Tethering PH- And Thermo-Responsive Polymer for ON-OFF Controlled Release of Guest Molecules. CrystEngComm 22(6) (2020) 1106-1111. https://doi.org/10.1039/c9ce01731c 10.1039/c9ce01731c

[95] Hu X. Lu Y. Dong C. Zhao W. Wu X. Zhou L. Chen L. Yao T. Shi S.. A RuII Polypyridyl Alkyne Complex Based Metal-Organic Frameworks for Combined Photodynamic/Photothermal/Chemotherapy. ChemistryA, European Journal 26(7) (2020) 1668-1675. https://doi.org/10.1002/chem.201904704 10.1002/chem.201904704 31814171

[96] Chen J. Liu J. Hu Y. Tian Z. Zhu Y.. Metal-Organic Framework-Coated Magnetite Nanoparticles for Synergistic Magnetic Hyperthermia and Chemotherapy with PH-Triggered Drug Release. Science and Technology of Advanced Material 20 (2019) 1043-1054. https://doi.org/10.1080/14686996.2019.1682467 10.1080/14686996.2019.1682467 PMC6844413 31723371

[97] Gharehdaghi Z. Naghib S.M. Rahimi R. Bakhshi A. Kefayat A. shamaeizadeh A. Molaabasi F.. Highly Improved PH-Responsive Anticancer Drug Delivery and T2-Weighted MRI Imaging by Magnetic MOF CuBTC-Based Nano/Microcomposite. Frontiers in Molecular Biosciences 10 (2023) 1071376. https://doi.org/10.3389/fmolb.2023.1071376 10.3389/fmolb.2023.1071376 37091862 PMC10114589

[98] Shahin R. Yousefi M. Ziyadi H. Bikhof M. Hekmati M.. PH-Responsive and Magnetic Fe3O4@UiO-66-NH2@PEI Nanocomposite as Drug Nanocarrier: Loading and Release Study of Imatinib. Inorganic Chemistry Communication 147 (2023) 110186. https://doi.org/10.1016/j.inoche.2022.110186 10.1016/j.inoche.2022.110186

[99] Lajevardi A. Hossaini Sadr M. Tavakkoli Yaraki M. Badiei A. Armaghan M.. A PH-Responsive and Magnetic Fe3O4@silica@MIL-100(Fe)/β-CD Nanocomposite as a Drug Nanocarrier: Loading and Release Study of Cephalexin. New Journal of Chemistry 42(12) (2018) 9690-9701. https://doi.org/10.1039/c8nj01375f 10.1039/c8nj01375f

[100] Tan L.L. Li H. Zhou Y. Zhang Y. Feng X. Wang B. Yang Y.W.. Zn2+-Triggered Drug Release from Biocompatible Zirconium MOFs Equipped with Supramolecular Gates. Small 11(31) (2015) 3807-3813. https://doi.org/10.1002/smll.201500155 10.1002/smll.201500155 25919865

[101] Rojas S. Hidalgo T. Luo Z. Ávila D. Laromaine A. Horcajada P.. Pushing the Limits on the Intestinal Crossing of Metal-Organic Frameworks: An Ex Vivo and in Vivo Detailed Study. ACS Nano 16 (2022) 5830-5838. https://doi.org/10.1021/acsnano.1c10942 10.1021/acsnano.1c10942 35298121 PMC9047668

[102] Zhou Y. Liu L. Cao Y. Yu S. He C. Chen X.. A Nanocomposite Vehicle Based on Metal-Organic Framework Nanoparticle Incorporated Biodegradable Microspheres for Enhanced Oral Insulin Delivery. ACS Applied Material & Interfaces 12 (2020) 22581-22592. https://doi.org/10.1021/acsami.0c04303 10.1021/acsami.0c04303 32340452

[103] Javanbakht S. Nezhad-Mokhtari P. Shaabani A. Arsalani N. Ghorbani M.. Incorporating Cu-Based Metal-Organic Framework/Drug Nanohybrids into Gelatin Microsphere for Ibuprofen Oral Delivery. Materials Science and Engineering C 96 (2019) 302-309. https://doi.org/10.1016/j.msec.2018.11.028 10.1016/j.msec.2018.11.028 30606537

[104] Zhou Y. Chen Z. Zhao D. Li D. He C. Chen X.. A PH-Triggered Self-Unpacking Capsule Containing Zwitterionic Hydrogel-Coated MOF Nanoparticles for Efficient Oral Exendin-4 Delivery. Advanced Materials 33(32) (2021) 2102044. https://doi.org/10.1002/adma.202102044 10.1002/adma.202102044 34216408

[105] Gao M. Yang C. Wu C. Chen Y. Zhuang H. Wang J. Cao Z.. Hydrogel-Metal-Organic-Framework Hybrids Mediated Efficient Oral Delivery of SiRNA for the Treatment of Ulcerative Colitis. Journal of Nanobiotechnology 20 (2022) 404. https://doi.org/10.1186/s12951-022-01603-6 10.1186/s12951-022-01603-6 36064365 PMC9446571

[106] Javanbakht S. Hemmati A. Namazi H. Heydari A.. Carboxymethylcellulose-Coated 5-Fluorouracil@MOF-5 Nano-Hybrid as a Bio-Nanocomposite Carrier for the Anticancer Oral Delivery. International Journal of Biolecular Macromolecules. 155 (2020) 876-882. https://doi.org/10.1016/j.ijbiomac.2019.12.007 10.1016/j.ijbiomac.2019.12.007 31805324

[107] Jiang K. Ni W. Cao X. Zhang L. Lin S.. A Nanosized Anionic MOF with Rich Thiadiazole Groups for Controlled Oral Drug Delivery. Matereials Today Bio 13 (2022) 100180. https://doi.org/10.1016/j.mtbio.2021.100180 10.1016/j.mtbio.2021.100180 PMC8649393 34927044

[108] Zou J.J. Wei G. Xiong C. Yu Y. Li S. Hu L. Ma S. Tian J.. Efficient Oral Insulin Delivery Enabled by Transferrin-Coated Acid-Resistant Metal-Organic Framework Nanoparticles. Science Advances 8(8) (2022) 4677. https://doi.org/10.1126/sciadv.abm4677 10.1126/sciadv.abm4677 PMC8865763 35196087

[109] Cheng G. Li W. Ha L. Han X. Hao S. Wan Y. Wang Z. Dong F. Zou X. Mao Y. Zheng S.Y.. Self-Assembly of Extracellular Vesicle-like Metal-Organic Framework Nanoparticles for Protection and Intracellular Delivery of Biofunctional Proteins. Journal of American Chemical Society 140 (2018) 7282-7291. https://doi.org/10.1021/jacs.8b03584 10.1021/jacs.8b03584 29809001

[110] Shang W. Peng L. Guo P. Hui H. Yang X. Tian J.. Metal-Organic Frameworks as a Theranostic Nanoplatform for Combinatorial Chemophotothermal Therapy Adapted to Different Administration. ACS Biomaterial Science & Engineering 6 (2020) 1008-1016. https://doi.org/10.1021/acsbiomaterials.9b01075 10.1021/acsbiomaterials.9b01075 33464845

[111] AbouAitah K. Higazy I.M. Swiderska-Sroda A. Abdelhameed R.M. Gierlotka S. Mohamed T.A. Szałaj U. Lojkowski W.. Anti-Inflammatory and Antioxidant Effects of Nanoformulations Composed of Metal-Organic Frameworks Delivering Rutin and/or Piperine Natural Agents. Drug Delivery 28 (2021) 1478-1495. https://doi.org/10.1080/10717544.2021.1949073 10.1080/10717544.2021.1949073 34254539 PMC8280904

[112] Zhang C. Hong S. Liu M.D. Yu X.Y. Zhang M.K. Zhang L. Zeng X. Zhang X.Z.. PH-Sensitive MOF Integrated with Glucose Oxidase for Glucose-Responsive Insulin Delivery. Journal of Controlled Release 320 (2020) 159-167. https://doi.org/10.1016/j.jconrel.2020.01.038 10.1016/j.jconrel.2020.01.038 31978443

[113] Luzuriaga M.A. Welch R.P. Dharmarwardana M. Benjamin C.E. Li S. Shahrivarkevishahi A. Popal S. Tuong L.H. Creswell C.T. Gassensmith J.J.. Enhanced Stability and Controlled Delivery of MOF-Encapsulated Vaccines and Their Immunogenic Response in Vivo. ACS Applied Material & Interfaces 11(10) (2019) 9740-9746. https://doi.org/10.1021/acsami.8b20504 10.1021/acsami.8b20504 30776885

[114] He M. Yu P. Hu Y. Zhang J. He M. Nie C. Chu X.. Erythrocyte-Membrane-Enveloped Biomineralized Metal-Organic Framework Nanoparticles Enable Intravenous Glucose-Responsive Insulin Delivery. ACS Applied Material & Interfaces 13(17) (2021) 19648-19659. https://doi.org/10.1021/acsami.1c01943 10.1021/acsami.1c01943 33890785

[115] Fernández-Paz C. Rojas S. Salcedo-Abraira P. Simón-Yarza T. Remuñán-López C. Horcajada P.. Metal-Organic Framework Microsphere Formulation for Pulmonary Administration. ACS Applied Material & Interfaces 12 (2020) 25676-25682. https://doi.org/10.1021/acsami.0c07356 10.1021/acsami.0c07356 32364369

[116] Hu X. Wang C. Wang L. Liu Z. Wu L. Zhang G. Yu L. Ren X. York P. Sun L. Zhang J. Li H.. Nanoporous CD-MOF Particles with Uniform and Inhalable Size for Pulmonary Delivery of Budesonide. International Journal of Pharmaceutics 564 (2019) 153-161. https://doi.org/10.1016/j.ijpharm.2019.04.030 10.1016/j.ijpharm.2019.04.030 30981874

[117] Zhou Y. Zhao Y. Niu B. Luo Q. Zhang Y. Quan G. Pan X. Wu C.. Cyclodextrin-Based Metal-Organic Frameworks for Pulmonary Delivery of Curcumin with Improved Solubility and Fine Aerodynamic Performance. International Journal of Pharmaceutics 588 (2020) 119777. https://doi.org/10.1016/j.ijpharm.2020.119777 10.1016/j.ijpharm.2020.119777 32805383

[118] Zhou Y. Niu B. Wu B. Luo S. Fu J. Zhao Y. Quan G. Pan X. Wu C.. A Homogenous Nanoporous Pulmonary Drug Delivery System Based on Metal-Organic Frameworks with Fine Aerosolization Performance and Good Compatibility. Acta Pharmaceutica Sinica B 10(12) (2020) 2404-2416. https://doi.org/10.1016/j.apsb.2020.07.018 10.1016/j.apsb.2020.07.018 33354510 PMC7745127

[119] Jarai B.M. Stillman Z. Attia L. Decker G.E. Bloch E.D. Fromen C.A.. Evaluating UiO-66 Metal-Organic Framework Nanoparticles as Acid-Sensitive Carriers for Pulmonary Drug Delivery Applications. ACS Applied Material & Interfaces 12 (2020) 38989-39004. https://doi.org/10.1021/acsami.0c10900 10.1021/acsami.0c10900 PMC7719435 32805901

[120] Zhou Y. Zhang M. Wang C. Ren X. Guo T. Cao Z. Zhang J. Sun L. Wu L.. Solidification of Volatile D-Limonene by Cyclodextrin Metal-Organic Framework for Pulmonary Delivery via Dry Powder Inhalers: In Vitro and in Vivo Evaluation. International Journal of Pharmaceutics 606 (2021) 120825. https://doi.org/10.1016/j.ijpharm.2021.120825 10.1016/j.ijpharm.2021.120825 34171430

[121] Márquez A.G. Hidalgo T. Lana H. Cunha D. Blanco-Prieto M.J. Álvarez-Lorenzo C. Boissière C. Sánchez C. Serre C. Horcajada P.. Biocompatible Polymer-Metal-Organic Framework Composite Patches for Cutaneous Administration of Cosmetic Molecules. Journal of Material Chemistry B 4 (2016) 7031-7040. https://doi.org/10.1039/c6tb01652a 10.1039/c6tb01652a 32263570

[122] Rojas S. Colinet I. Cunha D. Hidalgo T. Salles F. Serre C. Guillou N. Horcajada P.. Toward Understanding Drug Incorporation and Delivery from Biocompatible Metal-Organic Frameworks in View of Cutaneous Administration. ACS Omega 3 (2018) 2994-3003. https://doi.org/10.1021/acsomega.8b00185 10.1021/acsomega.8b00185 29623304 PMC5879486

[123] Rojas S. Horcajada P.. Understanding the Incorporation and Release of Salicylic Acid in Metal-Organic Frameworks for Topical Administration. European Journal of Inorganic Chemistry 14 (2021) 1325-1331. https://doi.org/10.1002/ejic.202001134 10.1002/ejic.202001134

[124] Bello M.G. Yang Y. Wang C. Wu L. Zhou P. Ding H. Ge X. Guo T. Wei L. Zhang J.. Facile Synthesis and Size Control of 2D Cyclodextrin-Based Metal-Organic Frameworks Nanosheet for Topical Drug Delivery. Particle and Particle Systems Characterization 37(11) (2020) 2000147. https://doi.org/10.1002/ppsc.202000147 10.1002/ppsc.202000147

[125] Gandara-Loe J. Souza B.E. Missyul A. Giraldo G. Tan J.C. Silvestre-Albero J.. MOF-Based Polymeric Nanocomposite Films as Potential Materials for Drug Delivery Devices in Ocular Therapeutics. ACS Applied Material & Interfaces 27 (2020) 30189-30197. https://doi.org/10.1021/acsami.0c07517 10.1021/acsami.0c07517 32530261

[126] Ahmadi M. Ayyoubzadeh S.M. Ghorbani-Bidkorbeh F. Shahhosseini S. Dadashzadeh S. Asadian E. Mosayebnia M. Siavashy S.. An Investigation of Affecting Factors on MOF Characteristics for Biomedical Applications: A Systematic Review. Heliyon 7(4) (2021) e06914. https://doi.org/10.1016/j.heliyon.2021.e06914 10.1016/j.heliyon.2021.e06914 33997421 PMC8100083

[127] Lawson H.D. Patrick Walton S. Chan C. Walton S.P. Lawson H.. Metal-Organic Frameworks for Drug Delivery: A Design Perspective. ACS Applied Materials & Interfaces 13(6) (2021) 7004-7020. http://dx.doi.org/10.1021/acsami.1c01089 10.1021/acsami.1c01089 33554591 PMC11790311

[128] Padmaa Paarakh M. Ani Jose P. Setty C.M. Christoper G.V.P.. Release kinetics - concepts and applications. International Journal of Pharmacy Research & Technology 8(1) (2019) 12-20. https://doi.org/10.31838/ijprt/2f08.01.02 10.31838/ijprt/2f08.01.02

[129] Horcajada P. Serre C. Maurin G. Ramsahye N.A. Balas F. Vallet-Regí M. Sebban M. Taulelle F. Férey G.. Flexible Porous Metal-Organic Frameworks for a Controlled Drug Delivery. Journal of American Chemical Society 130 (2008) 6774-6780. https://doi.org/10.1021/ja710973k 10.1021/ja710973k 18454528

[130] Esfahanian M. Ghasemzadeh M.A. Razavian S.M.H.. Synthesis, Identification and Application of the Novel Metal-Organic Framework Fe3O4@PAA@ZIF-8 for the Drug Delivery of Ciprofloxacin and Investigation of Antibacterial Activity. Artificial Cells, Nanomedicine, and Biotechnology 47 (2019) 2024-2030. https://doi.org/10.1080/21691401.2019.1617729 10.1080/21691401.2019.1617729 31112049

[131] Nasrabadi M. Ghasemzadeh M.A. Monfared M.R.Z.. The Preparation and Characterization of UiO-66 Metal-Organic Frameworks for the Delivery of the Drug Ciprofloxacin and an Evaluation of Their Antibacterial Activities. New Journal of Chemistry. 43(40) (2019) 16033-16040. https://doi.org/10.1039/c9nj03216a 10.1039/c9nj03216a

[132] Bazzazzadeh A. Dizaji B.F. Kianinejad N. Nouri A. Irani M.. Fabrication of Poly(Acrylic Acid) Grafted-Chitosan/Polyurethane/Magnetic MIL-53 Metal Organic Framework Composite Core-Shell Nanofibers for Codelivery of Temozolomide and Paclitaxel against Glioblastoma Cancer Cells. International Journal of Pharmaceutics 587 (2020) 119674. https://doi.org/10.1016/j.ijpharm.2020.119674 10.1016/j.ijpharm.2020.119674 32707243

[133] Leng X. Dong X. Wang W. Sai N. Yang C. You L. Huang H. Yin X. Ni J.. Biocompatible Fe-Based Micropore Metal-Organic Frameworks as Sustained-Release Anticancer Drug Carriers. Molecules. 23(10) (2018) https://doi.org/10.3390/molecules23102490 10.3390/molecules23102490 PMC6222375 30274195

[134] Xu Q. Li C. Chen Y. Zhang Y. Lu B.. Metal-Organic Framework-Based Intelligent Drug Delivery Systems for Cancer Theranostic: A Review. Frontiers of Materials Science 15 (2021) 374-390. https://doi.org/10.1007/s11706-021-0568-2 10.1007/s11706-021-0568-2

[135] Cai W. Wang J. Chu C. Chen W. Wu C. Liu G.. Metal-Organic Framework-Based Stimuli-Responsive Systems for Drug Delivery. Advanced Science 6(1) (2019) 1801526. https://doi.org/10.1002/advs.201801526 10.1002/advs.201801526 30643728 PMC6325578

[136] Velásquez-Hernández M.de J. Linares-Moreau M. Astria E. Carraro F. Alyami M.Z. Khashab N.M. Sumby C.J. Doonan C.J. Falcaro P.. Towards Applications of Bioentities@MOFs in Biomedicine. Coordination Chemistry Reviews 429 (2021) 213651. https://doi.org/10.1016/j.ccr.2020.213651 10.1016/j.ccr.2020.213651

